# Recruitment of dendritic cell progenitors to foci of influenza A virus infection sustains immunity

**DOI:** 10.1126/sciimmunol.abi9331

**Published:** 2021-11-05

**Authors:** Mar Cabeza-Cabrerizo, Carlos M. Minutti, Mariana Pereira da Costa, Ana Cardoso, Robert P. Jenkins, Justina Kulikauskaite, Michael D. Buck, Cecile Piot, Neil Rogers, Stefania Crotta, Lynne Whittaker, Hector Huerga Encabo, John W. McCauley, Judith E. Allen, Manolis Pasparakis, Andreas Wack, Erik Sahai, Caetano Reis e Sousa

**Affiliations:** 1 Immunobiology Laboratory, The Francis Crick Institute, 1 Midland Road, London NW1 1AT, UK; 2 Tumour Cell Biology Laboratory, The Francis Crick Institute, 1 Midland Road, London NW1 1AT, UK; 3 Immunoregulation Laboratory, The Francis Crick Institute, 1 Midland Road, London NW1 1AT, UK; 4 Worldwide Influenza Center, The Francis Crick Institute, 1 Midland Road, London NW1 1AT, UK; 5 Haematopoietic Stem Cell Laboratory, The Francis Crick Institute, 1 Midland Road, London NW1 1AT, UK; 6 School of Biological Sciences, Faculty of Biology, Medicine & Health, The University of Manchester, Oxford Rd, Manchester, M13 9PL, UK; 7 Institute for Genetics and Cologne Excellence Cluster on Cellular Stress Responses in Aging-Associated Diseases (CECAD), Joseph-Stelzmann-Str. 26, 50931 Cologne, Germany; 8 Current address: Centre for Misfolding Diseases, Yusuf Hamied Department of Chemistry, University of Cambridge, Lensfield Road, Cambridge CB2 1EW, UK

## Abstract

Protection from infection with respiratory viruses such as influenza A virus (IAV) requires T cell-mediated immune responses initiated by conventional dendritic cells (cDCs) that reside in the respiratory tract. Here, we show that effective induction of T cell responses against IAV in mice requires reinforcement of the resident lung cDC network by cDC progenitors. CCR2-binding chemokines produced during IAV infection recruit pre-cDCs from blood and direct them to foci of infection, increasing the number of progeny cDCs next to sites of viral replication. Ablation of CCR2 in the cDC lineage prevents this increase and results in a deficit in IAV-specific T cell responses and diminished resistance to re-infection. These data suggest that the homeostatic network of cDCs in tissues is insufficient for immunity and reveal a chemokine-driven mechanism of expansion of lung cDC numbers that amplifies T cell responses against respiratory viruses.

## Introduction

Conventional dendritic cells (cDCs) in tissues act as immune sentinels to detect viruses and microbes and migrate to draining lymph nodes to initiate innate and adaptive immune responses against pathogens (1). Although the number of cDCs in any given tissue is small, the cells can form a semi-continuous network at mucosal surfaces that allows surveillance of a large surface area, increasing the likelihood that pathogen entry will not go undetected. However, it is unknown whether this pool of resident cDCs in the tissue suffices for effective immunity or whether the cells need to increase in number at sites of infection in order to maximise local sampling of pathogen antigens and sustain induction of adaptive immune responses in lymph nodes. An inflammation-dependent increase in cDC numbers in tissues has been reported, including during corneal infection with herpes virus (2), chronic *Salmonella* infection (3), *Plasmodium* infection (4), cerebral ischemia and infection with pneumonia virus of mice or influenza A virus (IAV) (5–8). The latter is of particular interest as IAV is a major human pathogen and often used as a model for studying immunity to respiratory viruses (9).

cDCs are derived from proliferating pre-cDCs that leave the bone marrow (BM) and migrate via the blood to seed tissues (1, 10, 11). There, pre-cDC1s and pre-cDC2s terminally differentiate while maintaining residual mitotic activity, giving rise to small clones of sister cDC1s and cDC2s (7). The inflammation-dependent increase in tissue cDCs could reflect an increase in proliferative capacity but, in the case of IAV infection, proliferation does not account for local cDC expansion, which correlates instead with accelerated BM pre-cDC egress into the blood and an increase in pre-cDC influx into the lungs (7). Similarly, a population termed inflammatory cDC2s (inf-cDC2s), presumably derived from pre-cDC, was recently shown to be recruited to lungs after infection with pneumovirus in mice (5). Thus, inflammation can expand the network of tissue-resident cDCs by augmenting recruitment of blood-borne progenitors, which we have termed “emergency cDCpoiesis” (7). However, how pre-cDCs are recruited to infected lungs, whether they home to infection sites and the significance of emergency cDCpoiesis for antiviral immunity remain unclear.

Here, we set out to understand the mechanisms that underlie lung recruitment of pre-cDCs during IAV challenge and assess its impact on adaptive immunity to the virus. We report directional recruitment of pre-cDCs to specific foci of IAV infection in lungs. This directional recruitment is depended on pre-cDC expression of the chemokine receptor CCR2, which was upregulated in BM pre-cDCs upon IAV infection, enabling them to respond to CCR2 ligands. Of these ligands, we found that CCL2 is mainly produced by activated monocytes, which are positioned at infection foci and thereby contribute to reinforcement of the cDC network next to the antigen source. Notably, ablation of CCR2 expression in the cDC lineage selectively compromised expansion of the cDC network in IAV-infected lungs and limited the size of the migratory cDC (mig-cDC) population in lung draining lymph nodes (LNs). This resulted in diminished T cell responses to IAV, compromised establishment of a T cell memory population and reduced protection from re-infection. These findings suggest that the number of resident cDCs in tissues is insufficient for sustaining immune responses to respiratory virus infections in the absence of BM backup, highlighting the importance of emergency cDCpoiesis in immunity.

## Results

### Defining lung pre-cDCs by flow cytometry

**Figure 1:**
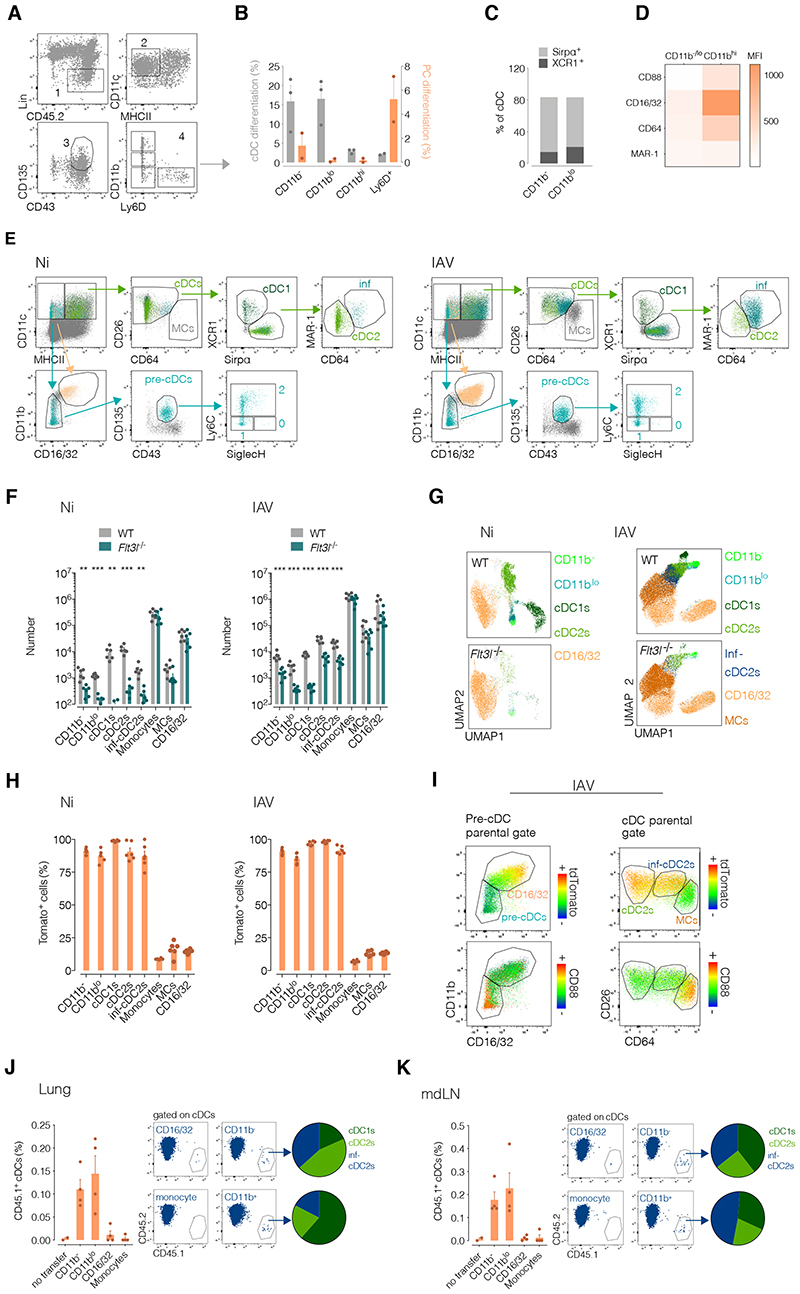
Defining the cDC lineage by flow cytometry in non-infected (Ni) and IAV-infected mice. (**A**) Live single cells from lung cell suspensions were analysed as follows: gate 1 on Lin^-^CD45^+^ cells (lineage includes anti-CD3, Ly6G, SiglecF, B220, CD19, NK1.1 and Ter119); gate 2 on CD11c^+^MHC-II^-^; gate 3 (pre-cDC gate) on CD135^+^CD43^+^. The bottom right panel shows CD11b and Ly6D staining in cells from the pre-cDC gate revealing 4 populations (gates 4): CD11b^-^ Ly6D^+^, CD11b^-^ Ly6D^-^, CD11b^lo^ Ly6D^-^ and CD11b^hi^ Ly6D^-^ cells. (**B**) The populations shown in gate 4, were sorted from the lungs of Ni C57BL/6 mice and cultured for 3 days with OP9-DL1 stromal cells in the presence of Flt3L. cDC- and PC-generating potential (a combination of cell recovery and subset differentiation) of these populations is shown in the left (see also Table S1). (**C**) cDC subset distribution from the experiment in B. cDC1s are defined as XCR1^+^ whereas cDC2s express SIRPα. (**D**) Heatmap comparing the expression of different monocyte markers, as measured by flow cytometry, in the CD11b^-/lo^ vs CD11b^hi^ gates 4 shown in (A). (**E**) Pre-cDC, cDC and CD11c^+^ MHC-II^+^ MC gating strategy in lung cell suspensions from naïve or IAV-infected mice. Panels shown have been pre-gated on single, live, CD45^+^ and lineage^-^ cells. The lineage cocktail includes antibodies against the following markers: CD3, Ly6G, SiglecF, B220, CD19, NK1.1, Ly6D and Ter119. Ly6C and SIglecH are used to identify pre-cDC subsets. Uncommitted pre-cDCs are SiglecH^+^ Ly6C^-^ (gate 0, bottom), pre-cDC1 are SiglecH^-^ Ly6C^-^ (gate 1, bottom) and pre-cDC2 are SiglecH^-/+^ Ly6C^+^ (gate 2, bottom). MCs are CD26^lo^ and CD64^hi^. CD26^hi^ and CD64^-/lo^ cDCs are divided into cDC1s (XCR1^+^) (gate 1, top), cDC2s (SIRPα^+^, CD64^-^) (gate 2, top) and inf-cDC2s (SIRPα^+^, CD64^int^) (gate inf, top). Coloring reflects backgating of the populations in question and arrows denote gate hierarchy. (**F**) Number of indicated myeloid cells per lung of naïve and IAV-infected (5dpi) WT vs. Flt3L-deficient mice. (**G**) Representative UMAPs of lung Lin^-^ CD11c^+^ cells from WT and Flt3L-deficient mice. (**H**) Tdtomato labelling of indicated myeloid cells in the lungs of naïve or IAV-infected (5dpi) *Clec9a^Cre^ R26^LSL-tdTomato^* mice. (**I**) Panels show TdTomato and CD88 (monocytic marker) fluorescence intensity in cells of the cDC lineage vs. unrelated cells. (**J**) TdTomato labelling of pre-cDCs (CD11b^-^ and CD11b^lo^), cDC1s, cDC2s, inf-cDC2s, monocytes and CD11c^+^ MHC-II^+^ MCs in the lungs of *Clec9a^Cre^ R26^LSL-tdTomato^* mice at 5 dpi with IAV. Panels show TdTomato and CD88 fluorescence intensity in cells of the cDC lineage vs. unrelated cells. Left: Percentage of CD45.1 cDCs recovered from lungs of CD45.2 recipient mice after transferring the CD45.1 cells listed on the x-axis. Middle: Reprentative dot plots. Right: Pie charts depicting cDC subsets recovered after transferring CD11b^-^ or CD11b^+^ pre-cDCs. (**K**) As in (J) but looking at mediastinal LN (mdLN). Each dot represents one mouse. Data in A-D is a pool of 2-3 experiments. F-K shows data pooled from two independent experiments. *t* test was used to compare WT and *Flt3l^-/-^* mice in F-G. * *p* ≤ 0.05, ** *p* ≤ 0.01, *** p ≤ 0.001. Not significant when not indicated.

To assess pre-cDC recruitment to the lungs in uninfected and IAV (X31 strain)-infected mice, we first validated strategies to identify the cells in lung cell suspensions by flow cytometry (12, 13). Mouse pre-cDCs are generally defined as leukocytes that are negative for many lineage-restricted markers (CD3, Ly6G, SiglecF, B220, CD19, NK1.1 and Ter119), as well as negative/low for surface expression of MHC class II, but positive for CD11c, CD135 and CD43 (Fig. 1A, gates 1-3) (14, 15). CD11b is often included as one of the lineage markers used as exclusion criteria (7, 12, 15). However, we found that the pre-cDC gate (Fig. 1A, gate 3) contained cells that expressed variable levels of CD11b, ranging from negative, low/intermediate to high levels (Fig. 1A, gates 4). We also found that the same pre-cDC gate contained cells that expressed Ly6D (Fig. 1A, gates 4), a marker of progenitors committed to giving rise to plasmacytoid cells (PCs) (16, 17).

To assess the cDC-generating potential of these populations, we FACS sorted them from the lungs of non-infected (Ni) C57BL/6 mice (Fig. 1A, gates 4: CD11b^-^ Ly6D^+^, CD11b^-^ Ly6D^-^, CD11b^lo^ Ly6D^-^ and CD11b^hi^ Ly6D^-^ cells) and cultured them on OP9-DL1 stromal cells for 3 days in the presence of Flt3L (18). We found that both the CD11b^-^ Ly6D^-^ and CD11b^lo^ Ly6D^-^ but not CD11b^-^ Ly6D^+^ or the CD11b^hi^ Ly6D^-^ gave rise to cDCs (Fig. 1B), comprising both cDC1 (XCR1^+^) and cDC2 (Sirpα^+^) subsets (Fig. 1C). The CD11b^-^ Ly6D^+^ population gave rise only to PCs whereas CD11b^hi^ Ly6D^-^ cells gave rise to neither (Fig. 1B). This suggested that a) Ly6D can be used to exclude pre-PCs from the pre-cDC gate, and b) pre-cDCs can express intermediate levels of CD11b, cautioning against the stringent use of that marker as a exclusion criterion. We therefore altered our lineage cocktail for pre-cDC definition to include anti-Ly6D and omit anti-CD11b. This approach failed to exclude from the pre-cDC gate a CD11b^hi^ population that expressed relatively high levels of many monocyte/macrophage markers, including CD16/32 (Fig. 1D). We therefore also used CD16/32 to help exclude CD11b^hi^ cells from the pre-cDC gate, leading to the gating strategy shown in Fig. 1E, starting from CD45^+^ Lin^-^ (CD3, Ly6G, Siglec-F, B220, CD19, NK1.1, Ly6D and Ter119) cells. This strategy allowed further sub-classification of pre-cDCs into uncommitted pre-cDCs (gate 0; Lin^-^, CD11c^+^ MHCII^-/low^, CD11b^-/low^ CD16/32^-^, CD135^+^ CD43^+^, Ly6C^-^ SiglecH^+^), pre-cDC1 (gate 1; Lin^-^, CD11c^+^ MHCII^-/low^, CD11b^-/low^ CD16/32^-^, CD135^+^ CD43^+^, Ly6C^-^ SiglecH^-^) and pre-cDC2 (gate 2; Lin^-^, CD11c^+^ MHCII^-/low^, CD11b^-/low^ CD16/32^-^, CD135^+^ CD43^+^, Ly6C^+^ SiglecH^-/+^) subsets as previously described (12, 13). In addition, we incorporated markers to allow for reliable separation of cDCs, inf-cDC2s, PCs, Ly6C^+^ monocytes and CD11c^+^ MHC-II^+^ monocyte-derived cells (MCs) (Fig. 1E, S1A), following the recently proposed guidelines (5).

### CD11b^-^, CD11b^lo^ and inf-cDC2s belong to the cDC lineage

We validated our cDC and pre-cDC identification strategy in three ways. First, we confirmed that pre-cDCs and cDCs, as identified by our gating strategy, were greatly reduced in mice lacking the essential cDC growth factor Flt3L (1), regardless of whether the mice were infected or not with IAV (Fig. 1F and G). In contrast, numbers of CD16/32^+^ (CD11c^+^ MHC-II^-^ CD11b^high^) cells that contaminate the pre-cDC gate, of MCs that fall in the CD11c^+^ MHC-II^+^ gate and of canonical Ly6C^+^ monocytes were the same in *Flt3l*-sufficient and -deficient mice (Fig. 1F and G). Second, using cDC lineage-tracing mice (*Clec9a^Cre/Cre^* × *Rosa26^tdTomato/tdTomato^*, abbreviated *Clec9a^tdTomato^*; (15)), we found that all pre-cDCs, cDC1s, cDC2s as defined by our gating strategy were equally labelled with tdTomato, confirming that they all belong to the cDC lineage (Fig. 1H). In contrast, Ly6C^+^ monocytes, CD16/32^+^ (CD11c^+^ MHC-II^-^ CD11b^high^) cells and CD11c^+^ MHC-II^+^ MCs were poorly labelled with tdTomato but expressed CD88, a marker recently proposed to allow identification of monocytic cells (5) (Fig. 1H, I). These observations held in IAV infected mice, which further revealed the recently-described CD64^+^ CD26^high^ CD88^-^ inf-cDC2 population to be tdTomato^+^, as expected from a *bona fide* cDC type (5) (Fig. 1H, I).

Third, to test the cDC-generating capacity of CD11b^lo^ and CD11b^-^ pre-cDC populations in an inflammatory setting, we sorted them (as indicated in Fig 1A, gates 4), as well as CD16/32^+^ (CD11c^+^ MHC-II^-^ CD11b^high^) cells (Fig. 1E) and Ly6C^+^ monocytes (Fig. S1A; used as a control), from the lungs of CD45.1 mice at 1 day post-infection (dpi) with IAV. We adoptively transferred the sorted cells i.v. into CD45.2 congenic mice infected 1 day earlier with IAV and analysed the recipient mice at 5 dpi when cDC lung numbers peak. We observed that CD11b^-^ and CD11b^lo^ pre-cDCs generated all cDC subsets, including inf-cDC2s, in both lungs and the mediastinal lymph nodes (mdLN) that drain the lung (Fig. 1J and K). In contrast, CD16/32^+^ (CD11c^+^ MHC-II^-^ CD11b^high^) cells and Ly6C^+^ monocytes did not give rise to cDC1s, cDC2s or inf-cDC2s (Fig. 1J and K) but to CD88^+^ CD64^+^ macrophage-like cells (Fig. S1B and C).

The latter were found predominantly in the lung (Fig. S1B and C), consistent the limited capacity of macrophage-like cells to migrate from lungs to lymph node during respiratory virus infection (5). In mdLNs, cells derived from transferred CD11b^-^ and CD11b^lo^ pre-cDCs contributed to both the resident and mig-cDC pool (Fig. S1D). Altogether, these data reassure that we can identify by flow cytometry most lung pre-cDCs and cDCs, including cells that have been previously ignored because of expression of CD11b or CD64. Furthermore, we confirm that CD11b^-^ and CD11b^lo^ pre-cDCs and inf-cDC2s are *bona fide* cDC lineage cells. CD11b^-^ and CD11b^lo^ pre-cDCs likely represent different stages of differentiation as suggested by their relative proximity to fully differentiated cDCs in UMAP analysis (Fig. 1G).

### cDC subsets increase in lungs of IAV-infected mice through increased recruitment of pre-cDCs

**Figure 2:**
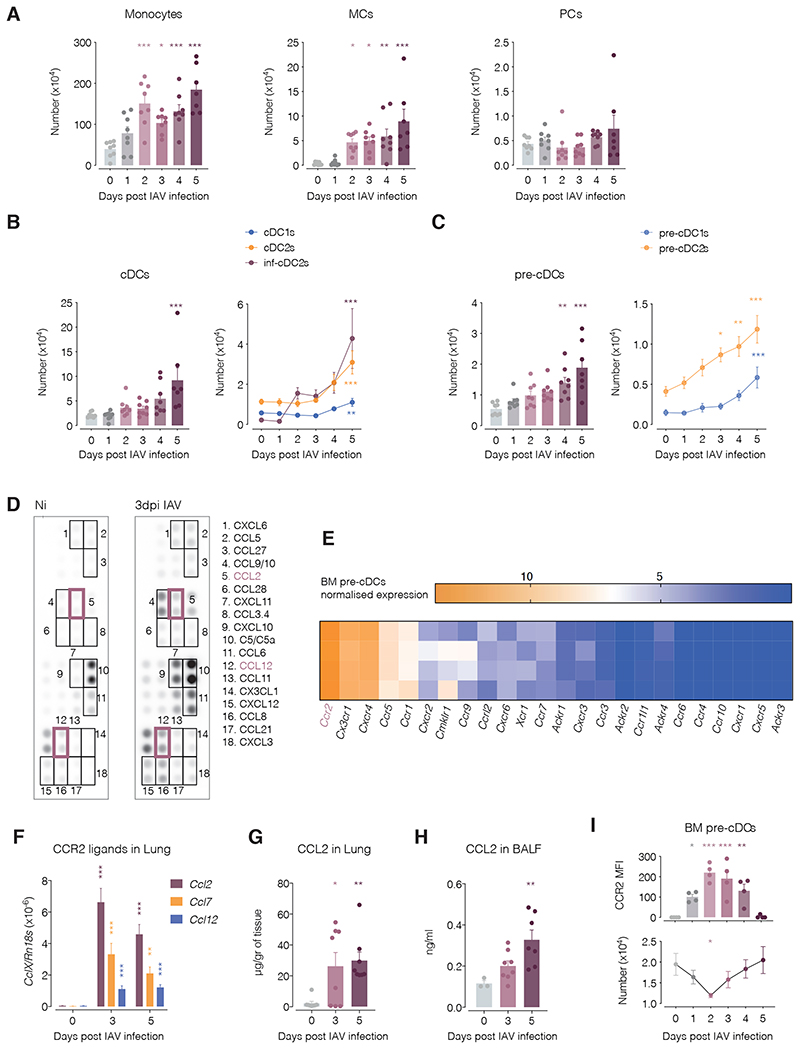
The lung cDC network expands during IAV infection. Flow cytometric determination of numbers of (**A**) Ly6C^+^ monocytes, CD11c^+^ MHC-II^+^ MCs and PCs, (**B**) cDCs and (**C**) pre-cDC subsets in lungs from mice infected with IAV. Cell populations were gated as described in Figure 1E and S2A. Each dot represents one mouse (*n* = 4-7). (**D**) Chemokine protein array showing the abundance of the indicated chemokines in BALF of a non-infected (upper panel) and an IAV-infected representative mouse at 3 dpi (lower panel). Black rectangles indicate duplicate blots in which an increase was observed between non-infected and infected mice. Purple rectangles indicate chemokines that are CCR2 ligands. Dots without rectangles are ones with either no visible changes or corresponding to control proteins from the array. (**E**) Normalised expression of chemokine receptor transcripts in sorted bone marrow pre-cDCs. Each row represents data from 1 mouse. (**F**) qPCR quantification of *Ccl2*, *Ccl12* and *Ccl7* transcripts from lung homogenates of Ni and IAV infected mice at different time points. CCL2 protein quantification in (**G**) lung homogenates (**H**) bronchoalveolar lavage fluid (BALF) after IAV infection. (**I**) CCR2 Mean Fluorescence Intensity (MFI) in BM pre-cDCs (top) or pre-cDC numbers (bottom) from Ni or IAV-infected mice at different time points. Each dot represents one mouse (*n* = 3-8) and data were pooled from 2 experiments. Cells were sorted using the gating strategy depicted in Fig. 1A, excluding the subset gating step. One-way ANOVA statistical test was used comparing day 0 (non-infected mice) to each day post-infection. * *p* ≤ 0.05, ** *p* ≤ 0.01, *** *p* ≤ 0.001. Not significant when not indicated.

After validating our flow cytometry strategy, we sought to analyse myeloid cell infiltration in lungs during IAV infection. Ly6C^+^ monocyte infiltration occurred early during infection, as expected (19), giving rise to an increase in MCs at 2 dpi (Fig. 2A). The number of PCs (gating strategy in Fig. S1A) did not change appreciably during the course of infection (Fig. 2A). In contrast, we observed a net increase in total lung cDCs and cDC subsets that became significant at 5 dpi (Fig. 2B) and was preceded by an earlier increase in total pre-cDCs and pre-cDC1/2 subsets at 4 and 5 dpi (Fig. 2C). Uncommitted pre-cDCs were almost absent from the lungs irrespective of infection (Fig. 1E), consistent with the notion that, even though such cells can be found in BM (12, 13), peripheral tissues are colonised predominantly by committed pre-cDC1s and pre-cDC2s (7). The increase in pre-cDCs and cDCs in the lung was mirrored in the lung mdLN (Fig. S2A) but not in the spleen (Fig. S2B). This tissue tropism suggests directional movement of pre-cDCs to the lung and lung-associated lymphoid tissues, likely guided by soluble mediators.

To investigate the latter point, we examined which chemokines were induced in the lung at 3 dpi, before pre-cDC recruitment is apparent. Using a semi-quantitative chemokine array, we found 17 chemokines increased in the bronchoalveolar lavage fluid (BALF) from infected animals compared to non-infected controls (Fig. 2D). To narrow down which might be relevant, we analysed the repertoire of chemokine receptors in pre-cDCs sorted from bone marrow and found highest expression of *Ccr2* transcripts, followed by *Cx3cr1* and *Cxcr4* (Fig. 2E), in line with previous observations (12). Mouse CCR2 recognises CCL2, CCL7 and CCL12, of which, CCL2 has the highest affinity for the receptor (20). *Ccl2*, *Ccl7* and *Ccl12* transcripts were all elevated in lung homogenates as measured by RT-qPCR (Fig. 2F) after IAV infection. CCL2 and CCL12 protein were represented in the chemokine array and elevated in BALF at 3 dpi (Fig. 2D). We confirmed that levels of CCL2 protein in lung tissue and BALF increased after infection, peaking at 5 dpi (Fig. 2G and H), the time of maximal pre-cDC influx (Fig. 2C). Interestingly, all BM pre-cDC subsets upregulated CCR2 after IAV infection, reaching highest intensity at 2dpi, which coincided with the peak of pre-cDC exit from the BM (Fig. 2I and S2C) (7). We therefore hypothesised that CCR2 mediates lung recruitment of pre-cDCs during IAV infection.

### Generation of a conditional CCR2 knockout mouse specific to the cDC lineage

To test this hypothesis, we crossed *Clec9^Cre/Cre^* mice (15) with *Ccr2^fl-eGFP/fl-eGFP^* mice (21). In progeny animals (*Clec9a^Cre/+^Ccr2^fl-eGFP/+^*), cells expressing *Clec9a* recombine the *Ccr2* locus to excise exon 3 from the *Ccr2* gene, allowing expression of enhanced green fluorescent protein (eGFP), which reports on targeted cells (Fig. S3A). Mice carrying two copies of the engineered *Ccr2* locus generate a non-functional truncated CCR2 protein in Cre-expressing cells (21). Therefore, *Clec9a^Cre/+^Ccr2^fl-eGFP/fl-eGFP^* (here termed *Clec9a^ΔCCR2^* or *C9a^ΔCCR2^*) can be used to ablate CCR2 in *Clec9a*-expressing cells and compared to *Clec9a^Cre/+^Ccr2^fl-eGFP/+^* (here termed *Clec9a^WTCCR2^* or *C9a^WTCCR2^*) littermates whose *Clec9a*-expressing cells express normal levels of CCR2 because the locus is haplosufficient (22).

**Figure 3:**
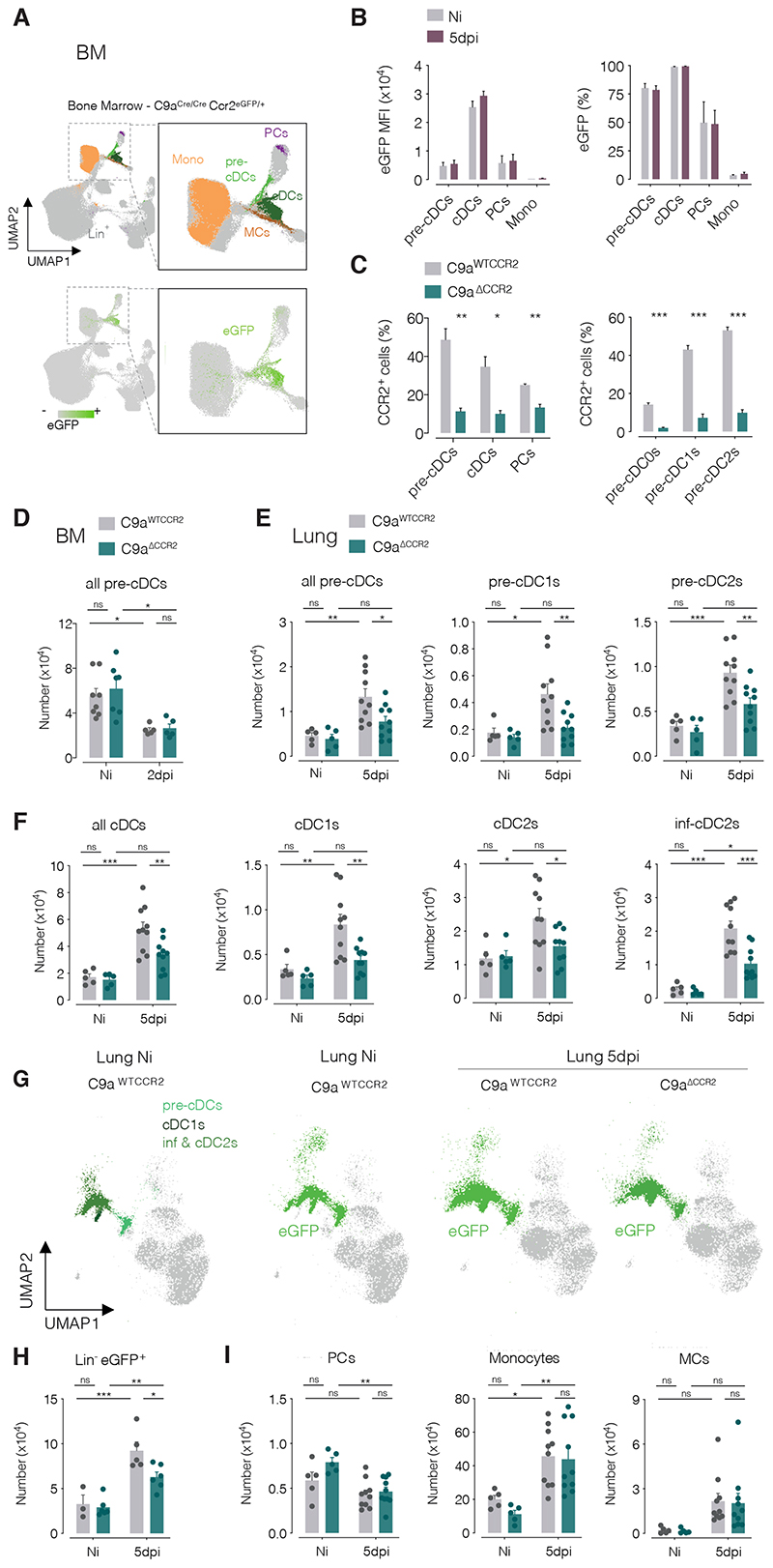
Expansion of the cDC network during IAV infection is CCR2 dependent. (**A**) UMAP analysis of flow cytometry data showing CD45^+^ cells overlaid with different immune populations as identified with manual gating (top panel) and eGFP intensity (bottom panel) in BM of a *C9a^Cre/Cre^ Ccr2^fl-eGFP/+^* mouse at steady state. (**B**) eGFP MFI (left) and percentage eGFP^+^ cells (right) for different cell populations from the BM of *C9a^Cre/Cre^ Ccr2^fl-eGFP/fl-eGFP^* mice uninfected (grey) or infected with IAV (purple). (**C**) CCR2^+^ cells in different populations from the BM of *C9a^WTCCR2^* (grey) or *C9a^ΔCCR2^* (teal) mice at 2 dpi with IAV. (**D**) Number of BM pre-cDCs in *C9a^WTCCR2^* (grey) or *C9a^ΔCCR2^* (teal) Ni or 2dpi IAV-infected mice (**E**) Numbers of pre-cDCs and (**F**) cDCs in lungs from naïve and 5dpi IAV-infected *C9a^WTCCR2^* (grey) or *C9a^ΔCCR2^* (teal) mice. (**G**) UMAP analysis of flow cytometry data showing Lin^-^ CD11b^low^ cells overlaid with different immune populations as identified with manual gating (left panel) and eGFP positive cells overlayed in samples from the lungs of naïve and infected *C9a^WTCCR2^* or *C9a^ΔCCR2^* mice as indicated. (**H**) Quantification of Lin^-^ eGFP^+^ cells in lungs from *C9a^WTCCR2^* (grey) or *C9a^ΔCCR2^* (teal) mice Ni or 5dpi with IAV. Lineage was defined as in Figure 1. (**I**) Numbers of PCs, Ly6C^+^ monocytes and CD11c^+^ MHC-II^+^ MCs in lungs from naïve and 5dpi IAV-infected *C9a^WTCCR2^* (grey) or *C9a^ΔCCR2^* (teal) mice. Each dot in (B-F, H, I) represents one mouse (*n* = 5-10) and data were pooled from 2 experiments. Statistical analysis was done using a two-way ANOVA test comparing infected *C9a^WTCCR2^* and *C9a^ΔCCR2^* mice. * *p* ≤ 0.05, ** *p* ≤ 0.01, *** *p* ≤ 0.001. Not significant (ns).

*Clec9a*-expressing cells include cDC1s, PCs, conventional DC progenitors (CDPs) and pre-cDCs (7, 15). To identify targeted cells in *Clec9a^WTCCR2^* and *Clec9a^ΔCcr2^* mice, we analysed the myeloid compartments of BM and lung for eGFP^+^ cells. Pre-cDCs and cDCs were prominent among labelled cells in the BM, both in frequency (> 75% penetrance) and mean fluorescence intensity (Fig. 3A and B), which remained unaltered by IAV infection (Fig. 3B and S3B). As expected, monocytes were not labelled (Fig. 3A, B). Consistent with these results, a large reduction in CCR2 positivity, at its peak of expression in pre-cDCs (2dpi; Fig. 2I), was seen in all BM pre-cDC (Fig. 3C) and lung cDC subsets (Fig. S3C) from *Clec9a^ΔCCR2^* mice when compared to the same cells in *Clec9a^WTCCR2^* controls. In contrast, expression of CCR2 by PCs (Fig. 3C) or monocytes (Fig. S3D) was not significantly altered in *Clec9a^ΔCCR2^* mice vs *Clec9a^WTCCR2^* controls. Further, CCR2-dependent exit of monocytes from BM was similar in the two strains, both at steady-state and after IAV infection (Fig. S3D). We conclude that *Clec9a^WTCCR2^* mice allow eGFP marking of pre-cDCs and cDCs while *Clec9a^ΔCCR2^* mice constitute a useful tool to selectively ablate CCR2 expression in the cDC lineage.

### CCR2 mediates recruitment of pre-cDCs to infected lungs

Comparison of uninfected *Clec9a^WTCCR2^* and *Clec9a^ΔCCR2^* mice revealed a similar number of pre-cDCs and cDCs in the BM and lungs (Fig. 3D-F). This is in line with a report that CCR2 is dispensable for lung seeding by pre-cDCs at steady state (23) and consistent with the low levels of CCR2 ligands in that organ in the absence of inflammation (Fig. 2F, G). We next asked next whether the reduction in CCR2 expression in pre-cDCs from *Clec9a^ΔCCR2^* mice affects their lung recruitment upon IAV infection. Interestingly, CCR2 ablation in *Clec9a^ΔCCR2^* mice did not affect pre-cDC BM exit after IAV infection, as BM pre-cDCs were equally reduced in mice of both genotypes (Fig. 3D). However, at 5dpi, we observed significantly fewer total pre-cDCs, including pre-cDC1 and pre-cDC2 subsets, in lungs of *Clec9a^ΔCCR2^* mice compared to *Clec9a^WTCCR2^* controls (Fig. 3E). This correlated with reduced infection-induced expansion in lung cDC1, cDC2 and inf-cDC2 numbers in *Clec9a^ΔCCR2^* mice (Fig. 3F). Dimensionality reduction analysis of flow cytometry data confirmed that eGFP^+^ cells lacking lineage-restricted markers (CD3, Ly6G, SiglecF, B220, CD19, NK1.1, Ly6D and Ter119) expressing low levels of CD11b (corresponding to pre-cDCs and cDCs) increased to a smaller extent in lungs of infected *Clec9a^ΔCCR2^* mice compared to *Clec9a^WTCCR2^* controls (Fig. 3G, H). This could not be explained by changes in eGFP labelling during infection as BM pre-cDCs and lung pre-cDCs were equally targeted in both genotypes regardless of infection status (Fig. 3B). PCs, Ly6C^+^ monocytes and CD11c^+^ MHC-II^+^ MCs were found in equal numbers in the lungs of uninfected *Clec9a^WTCCR2^* and *Clec9a^ΔCCR2^* mice (Fig. 3I). Importantly, the latter two cell populations markedly increased in abundance after infection irrespective of mouse genotype (Fig. 3I), confirming that monocyte mobilization is not affected in *Clec9a^ΔCCR2^* mice.

When examining the lung draining mdLNs, we observed that CCR2 ablation in *Clec9a^ΔCCR2^* mice also compromised the infection-associated increase in mig-cDCs (Fig. S3E). This is likely an indirect consequence of the decreased recruitment of *Clec9a^ΔCCR2^* pre-cDCs to infected lungs, which results in fewer differentiated cDCs able to emigrate via afferent lymph to mdLNs, a process driven by CCR7. To establish this point (and exclude a direct effect of CCR2 ablation on the ability of lung cDC to migrate from lung to mdLNs), we measured surface expression of CCR2, CCR7 and the activation marker CD86 on lung cDCs at 3dpi, around the point of maximal cDC migration to mdLN. As expected, all cDC subsets upregulated CD86 upon IAV infection (Fig. S3F). Cells poised to emigrate also upregulated CCR7 and a clear fraction of cDC1, cDC2 and inf-cDC2 was positive for CCR7 in lungs from IAV-infected animals (Fig. S3G, H). Importantly, CCR7^+^ cells downregulated CCR2 (Fig. S3G, H), indicating that CCR7 and CCR2 expression in cDC subsets are mutually exclusive and implying that the CCR2 axis might actually oppose cDC migration to lymph nodes. Supporting this notion, we observed that induction of CCR2 ligands in mdLN was much lower than in lungs (Fig. S3I), suggesting that infection induces a CCR2 ligand gradient that is high in the lungs and lower in mdLN, thereby retaining CCR2-expressing cells in the infected tissue rather than promoting their migration to draining mdLNs.

Finally, we tested whether CCR2 was required for pre-cDC homing to the lungs in different types of inflammation. We infected *Clec9a^WTCCR2^* and *Clec9a^ΔCCR2^* mice with *Nippostrongylus brasiliensis*, a lung-migrating nematode that induces acute tissue injury followed by a strong type 2 inflammatory response (24). Like IAV, parasite infection induced pre-cDC recruitment to lungs (Fig. S4A) but, in contrast to infection with the virus, this was unaffected by loss of CCR2 in cDC precursors (Fig. S4B).

Overall, these data suggest that, during IAV infection, pre-cDCs in blood follow a gradient of CCR2 ligands to find their way into IAV-infected lungs where they expand the network of cDC1s, cDC2s and inf-cDC2s. Those cDCs can then become activated to downregulate CCR2 and upregulate CCR7 in order to migrate to draining mdLNs, presumably carrying IAV antigens.

### Clec9a^Cre^Ccr2^fl-eGFP^ mice can be used to visualise pre-DCs by microscopy

**Figure 4:**
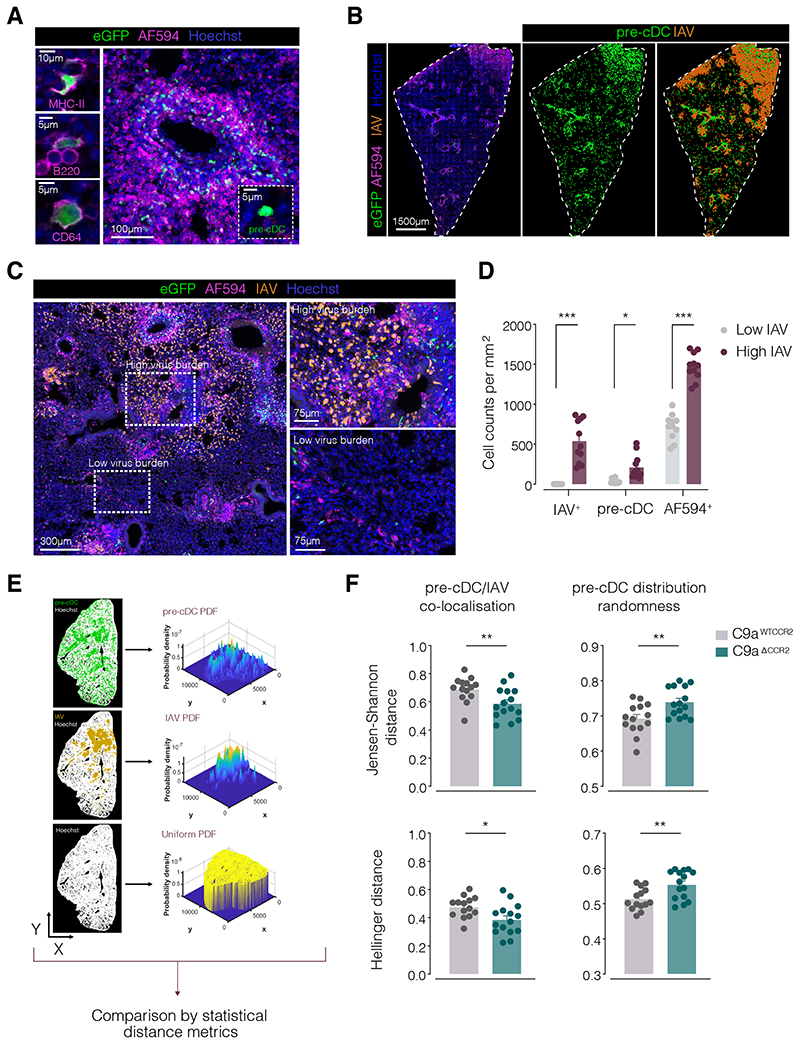
Pre-cDCs are specifically recruited to foci of IAV infection in a CCR2-dependent manner. (**A**) Single z optical slices of *C9a^Cre/+^ Ccr2 ^fl-eGFP/+^* mouse lung sections stained with AF594-conjugated anti-MHC class II (upper left), anti-B220 (middle left), anti-CD64 (bottom left) or with a cocktail (right; 3D projection) of all three antibodies (AF594). Images on the left are examples of eGFP^+^ cells that co-stain for each marker. Image on the bottom right depicts an example of an eGFP^+^ cell that does not co-stain for AF594 and corresponds to a pre-cDC (inset). Hoechst was used to visualize cell nuclei. (**B**) 3D projection of a whole *C9a^Cre/+^ Ccr2 ^fl-eGFP/+^* mouse lung section 5 days post infection with IAV stained with Hoechst, anti-IAV M+NP and for AF594 exclusion markers (anti-MHC class II, anti-CD64, anti-B220) (left). Middle panel shows the localization of pre-cDCs (eGFP^+^ AF594^−^) and right panel that of IAV^+^ cells (M+NP staining) based on the coordinates of volumetric surfaces generated using Imaris software. Dashed lines delineate the outline of imaged lung tissue based on Hoechst staining. (**C**) 3D projection of a *C9a^Cre/+^ Ccr2 ^fl-eGFP/+^* mouse lung section 5 days post infection with IAV stained as in (B). Dashed rectangles and insets highlight regions of high and low staining for IAV M + NP. (**D**) Quantification of IAV M+NP^+^ cells, pre-cDCs and AF594^+^ (MHC-II, B220 and CD64) cells in volumes of high (purple) and low (grey) IAV burden (defined as in C). (**E**) Workflow for the analysis of pre-cDC distribution in IAV-infected lungs, including the generation of probability density functions (PDFs) corresponding to pre-cDC, IAV M+NP^+^ and Hoechst staining. (**F**) Pre-cDC vs. IAV M+NP co-localization (left) and pre-cDC randomness (right) scores for *C9a^WTCCR2^* (grey) and *C9a^ΔCCR2^* (teal) IAV-infected mice calculated by Jensen-Shannon (upper panels) and Hellinger (lower panels) distance metrics. Pre-cDC vs. IAV co-localisation was determined by comparison of pre-cDC and IAV PDFs. Pre-cDC distribution randomness was determined by comparison of pre-cDC PDF and Hoechst PDF. Each dot corresponds to the analysis of one image from a lung section (*n* = 29 images from 10 mice). Statistical analyses were performed using unpaired t-test. * *p* ≤ 0.05, ** *p* ≤ 0.01, *** *p* ≤ 0.001.

IAV infection of the lung is known to be heterogeneous and virally-infected cells remain localised to discrete foci (7, 25). We wondered whether recruited pre-cDCs were directed to such foci. We confirmed that most pre-cDCs arriving in lungs of *Clec9a^Cre^Ccr2^fl-eGFP^* mice express eGFP (Fig. S5A), as noted in BM (Fig. 3B). We therefore used this eGFP expression to visualise pre-cDCs *in situ* in lung tissue sections from *Clec9a^Cre^Ccr2^fl-eGFP^* mice, excluding any cells that stained with AF594-conjugated anti-MHC class II, anti-B220 and anti-CD64 antibodies (AF594^+^ cells) to eliminate eGFP^+^ cDCs, PCs, monocytes and macrophages from our analysis (Fig. 4A; S5B, C). Co-staining for IAV matrix (M) and nucleoprotein (NP) identified the foci of infection, and visual inspection suggested that they contained higher numbers of pre-cDCs than adjacent areas (Fig. 4B, C). This was confirmed by quantitating pre-cDC numbers in defined lung 3D volumes with high vs low virus burden (Fig. 4D). Volumes with high virus burden also contained more MHC-II^+^ CD64^+^ and/or B220^+^ cells irrespective of eGFP marking (Fig. 4D; referred to as AF594^+^), likely corresponding to inflammatory cells, such as CD64^high^ monocytes and MHC-II^+^ cDCs.

### CCR2 specifically recruits pre-cDCs to infection foci in the lung

We then tested whether CCR2 is necessary for pre-cDC localisation at infection foci by measuring co-localisation between pre-cDC surfaces (eGFP^+^ MHC-II^-^ CD64^-^ B220^-^) and IAV-infection foci (M^+^ NP^+^) in *Clec9a^WTCCR2^* versus *Clec9a^ΔCCR2^* lung slices (Fig. 4E). We used a form of statistical analysis that is robust within the range of population sizes observed in images and therefore unaffected by lower pre-cDC numbers in the infected lungs of *Clec9a^ΔCcr2^* mice (Fig. 4E). Notably, the co-localisation score of pre-cDCs and IAV was consistently higher in *Clec9a^WTCCR2^* compared to *Clec9a^ΔCCR2^* lung sections when quantified by two statistical distance metrics (Jensen-Shannon and Hellinger distances; Fig. 4F). Conversely, using the same distance metrics, the “randomness” of pre-cDC positioning (measured by comparison of actual pre-cDC distribution to a theoretical distribution uniformly spread across the whole tissue) was greater in *Clec9a^ΔCCR2^* compared to *Clec9a^WTCCR2^* lungs (Fig. 4F) even though the infection foci were positioned to an equally random extent between genotypes (Fig. S5D, E). In contrast to pre-cDCs, there was no difference between the two mouse strains in the number of AF594^+^ cells in regions of high or low IAV infiltration (Fig. S5F). These data suggest that CCR2 is used by pre-cDCs not only to migrate into IAV-infected lungs but specifically to home to the areas of active infection.

### Ccl2 is predominantly expressed by activated monocytes during IAV infection

**Figure 5:**
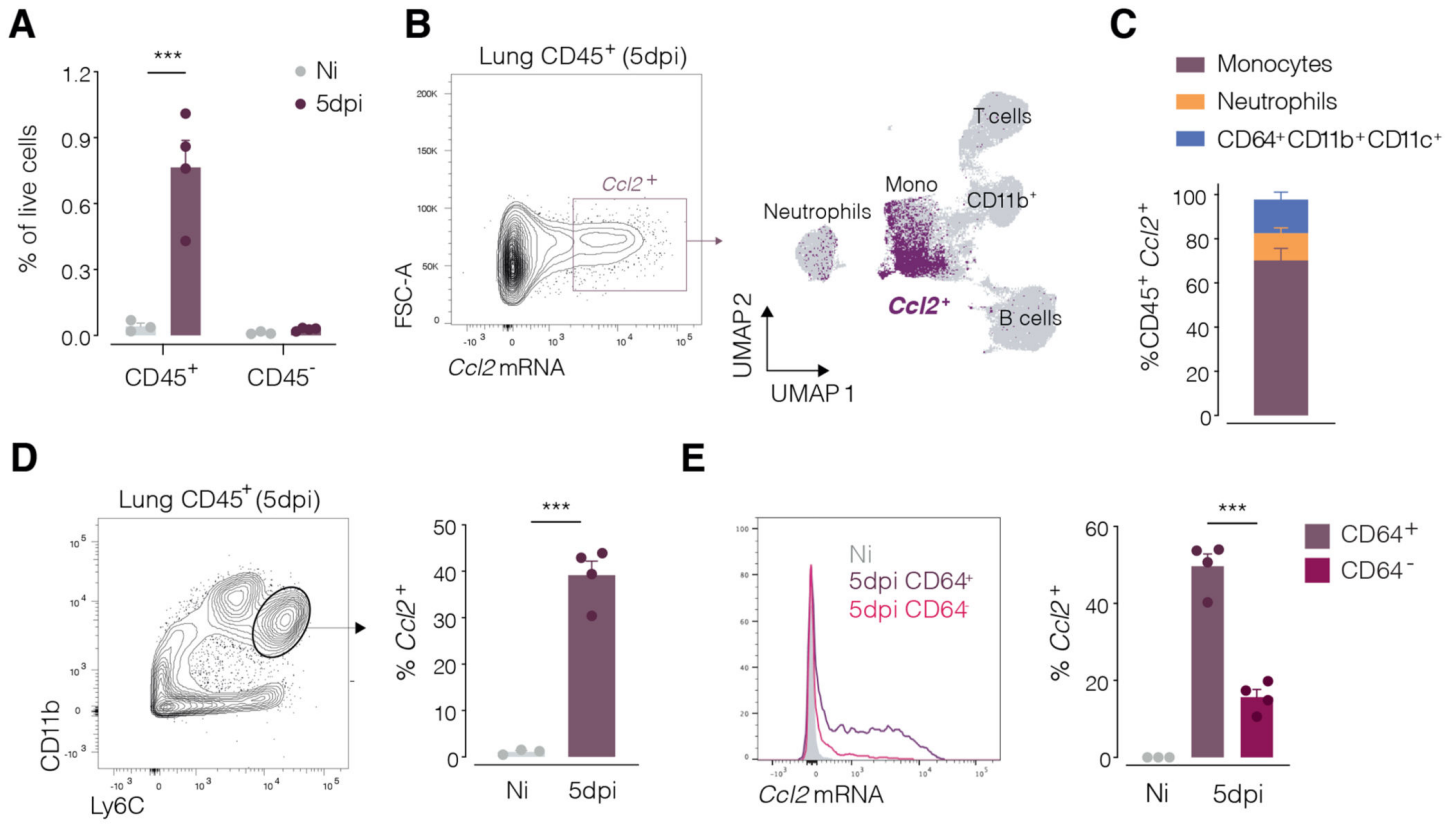
Activated monocytes are the main source of *Ccl2* transcripts during IAV infection PrimeFlow analysis of *Ccl2* transcripts in lung cells from non-infected and IAV-infected mice (5dpi). (**A**) CD45^+^ and CD45^-^ lung cells as a percentage of live *Ccl2^+^* cells (**B**) UMAP analysis of flow cytometry data indicating clusters of immune cell populations from a mouse lung 5 days post IAV infection, overlaid with *Ccl2^+^* cells (purple) gated as indicated in the representative plot on the left. (**C**) Composition of lung *Ccl2^+^* cells 5 days post IAV infection (*n* = 4 mice). (**D**) Percentage of lung *Ccl2^+^* monocytes (CD11b^+^ Ly6C^hi^) in non-infected and infected mice (5dpi) gated as indicated in the representative plot on the left. (**E**) Histogram showing modal intensity of *Ccl2* mRNA PrimeFlow probe in CD11b^+^ Ly6C^hi^ monocytes from non-infected mice (grey) and from CD64^+^ (teal) and CD64^-^ (purple) monocytes. Plot shows percentage of *Ccl2^+^* monocytes in CD64^+^ and CD64^-^ monocyte populations in infected mice (5dpi) or in total monocytes in non-infected mice. Each dot represents one mouse (*n* = 3-4) and data shows 1 representative experiment out of 3. Statistical analysis was done using a two-way ANOVA in A and one-way ANOVA in D, E. *** p ≤ 0.001. Not significant when not indicated.

To investigate sources of CCR2 ligands that might underlie recruitment of pre-cDCs to infection foci, we used PrimeFlow analysis to quantify *Ccl2* transcripts by flow cytometry. At 5dpi, more than 90% of *Ccl2^+^* cells were of hematopoietic origin (CD45^+^) (Fig. 5A), in contrast with earlier time points at which CCL2 is produced by CD45^−^epithelial cells (19). Among CD45^+^ *Ccl2*-expressing cells, the majority corresponded to Ly6C^+^ monocytes (Fig. 5B, C), which are recruited earlier than pre-cDCs during IAV infection (Fig. 2A). This is consistent with the notion that monocytes recruited to inflammatory sites can produce CCL2 in a positive feedback amplification loop that recruits additional cells (26). Around 40% of Ly6C^+^ monocytes expressed *Ccl2* at 5dpi (Fig. 5D) but there was a noticeable skew in staining towards monocytes with higher CD64 expression (Fig. 5E). CD64^+^ monocytes correspond to those with a more activated phenotype (27) and might explain the observation that pre-cDCs co-localise at foci of infection with higher numbers of MHC-II, B220 and CD64-positive cells (see above; Fig. 4D). Overall, these data suggest that early recruited monocytes, activated adjacent to IAV infection foci, produce CCL2 that attracts not only additional monocytes but also pre-cDCs.

### Acute T cell responses are impaired in Clec9a^ΔCCR2^ mice

**Figure 6:**
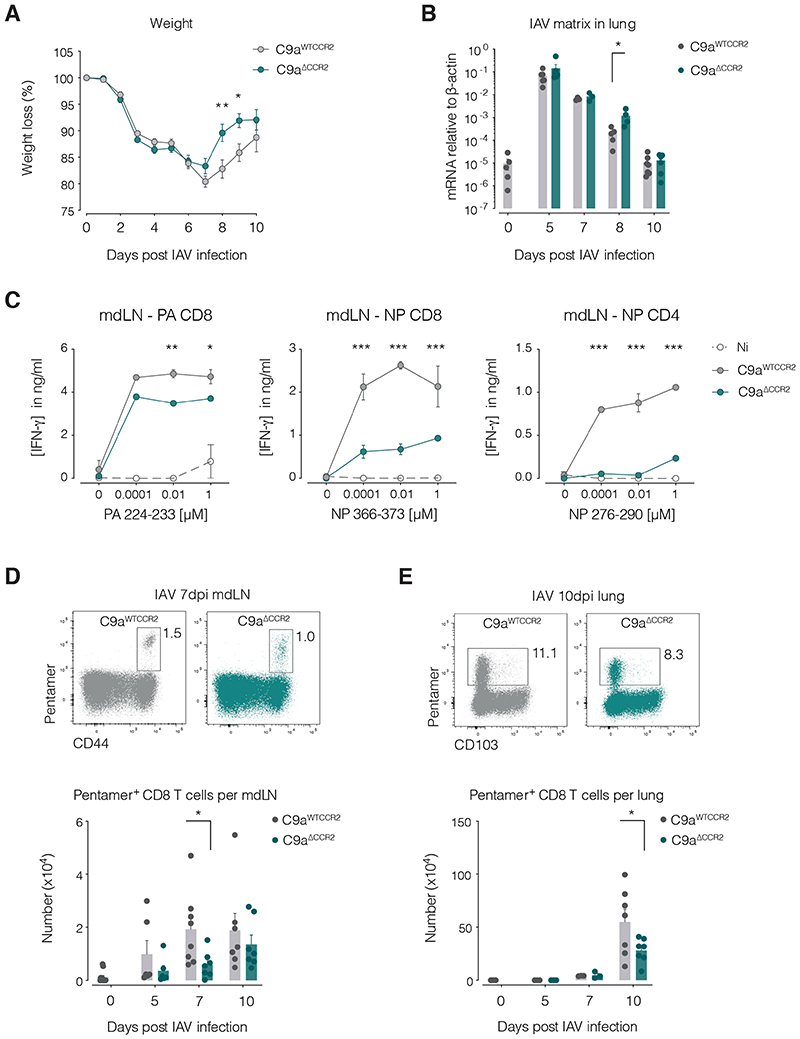
IAV-specific T cell responses are impaired in *C9a^ΔCCR2^* mice. (**A**) Weight curves for *C9a^WTCCR2^* (grey) and *C9a^ΔCCR2^* (teal) mice infected with IAV. Data are mean from 7 mice pooled from 2 experiments. Error bars represent SEM. (**B**) RT-qPCR for mRNA encoding IAV matrix protein in lungs of *C9a^WTCCR2^* (grey) and *C9a^ΔCCR2^* (teal) mice at the indicated dpi. Each dot represents one mouse (*n* = 4-6) and data were pooled from 3 experiments. (**C**) Quantification of IFN-γ in supernatants following 72h of *ex vivo* re-stimulation of mdLN cells from infected *C9a^WTCCR2^* (grey, continuous) or *C9a^ΔCCR2^* (teal) mice or Ni controls (open circles, dotted) with the indicated doses of IAV PA or NP peptides recognized by IAV-specific H-2^b^-restricted CD8^+^ or CD4^+^ T cells. Data in peptide re-stimulation treatments are mean from 2-3 mice from 1 experiment out of 2. Flow cytometry analysis of the number of IAV-specific CD8^+^ T cells per mediastinal lymph node (mdLN) (**D**) or lungs (**E**) of *C9a^WTCCR2^* and *C9a^ΔCCR2^* mice at the indicated time points post infection with IAV. Data show responses in *C9a^WTCCR2^* (grey) or *C9a^ΔCCR2^* (teal) mice at the specified time points after IAV infection with non-infected (Ni) mice serving as specificity controls. Each dot represents one mouse (*n* = 7-10) and data were pooled from 2 experiments. CD8 T cells were gated as live, CD45^+^ cells, B220^-^, TCRβ^+^, CD8^+^ and CD4^-^. Statistical analysis was done using a two-way ANOVA test in A, C and one-way ANOVA test in B, D, E comparing infected *C9a^WTCCR2^* and *C9a^ΔCCR2^* mice. * *p* ≤ 0.05, ** *p* ≤ 0.01, *** *p* ≤ 0.001. Not significant when not indicated.

To assess the immunological relevance of these observations, we examined T cell priming and viral clearance in *Clec9a^WTCCR2^* versus *Clec9a^ΔCCR2^* mice. In uninfected mice, we did not observe any differences in immune cell composition in spleen and lung draining mediastinal LNs (mdLNs) between *Clec9a^ΔCCR2^* vs *Clec9a^WTCCR2^* mice (Fig. S6A–D). However, upon IAV infection, *Clec9a^ΔCCR2^* mice displayed less weight loss at 8 and 9 d.p.i compared to controls (Fig. 6A), a timepoint that coincided with higher abundance of mRNA encoding IAV matrix protein in their lungs (Fig. 6B). These two observations are consistent with the possibility that *Clec9a^ΔCCR2^* mice mount an impaired T cell response to IAV, which reduces their ability to clear the virus at late infection timepoints but spares them from T cell-induced immunopathology causing weight loss (28).

To examine this in more detail, we re-stimulated cell suspensions taken from mdLNs or infected lungs with peptides corresponding to known H-2^b^ MHC class I- or II-restricted IAV epitopes. Cultures of mdLN or lung cells from *Clec9a^ΔCCR2^* mice accumulated less IFN-γ than those from *Clec9a^WTCCR2^* controls in response to IAV peptides (Fig. 6C, S7A). Consistent with those findings, the frequency and number of IAV NP-specific H-2D^b^-restricted CD8^+^ T cells were reduced in mdLNs and lungs from *Clec9a^ΔCCR2^* mice compared to *Clec9a^WTCCR2^* controls at 7 and 10 dpi (Fig. 6D, E). In contrast, IgM and IgG antibody responses to IAV were unaffected in *Clec9a^ΔCCR2^* mice (Fig. S7B). To rule out an intrinsic defect in T cell priming in *Clec9a^ΔCCR2^* mice, we measured T cell responses after infection with *N*. *brasiliensis* and found that they were similar to those in control mice (Fig. S7C). Therefore, the defect in the T cell response to IAV likely reflects the fact that, in the absence of cDC network expansion around virus infection foci, the number of activated mig-cDCs carrying IAV antigens from lung to mdLNs becomes limiting and, as a consequence, T cell priming is compromised (29). Consistent with this notion, at 5dpi there was a smaller population of mig-cDCs in mdLNs from infected *Clec9a^ΔCCR2^* mice compared to controls (Fig. S3E) and they appeared less activated as measured by CD86 expression (Fig. S7D). These data suggest that acute T cell responses against IAV are curtailed if lung cDC numbers are not sufficiently boosted via CCR2-mediated pre-cDC recruitment.

### Clec9a^ΔCCR2^ mice are more susceptible to IAV reinfection

**Figure 7:**
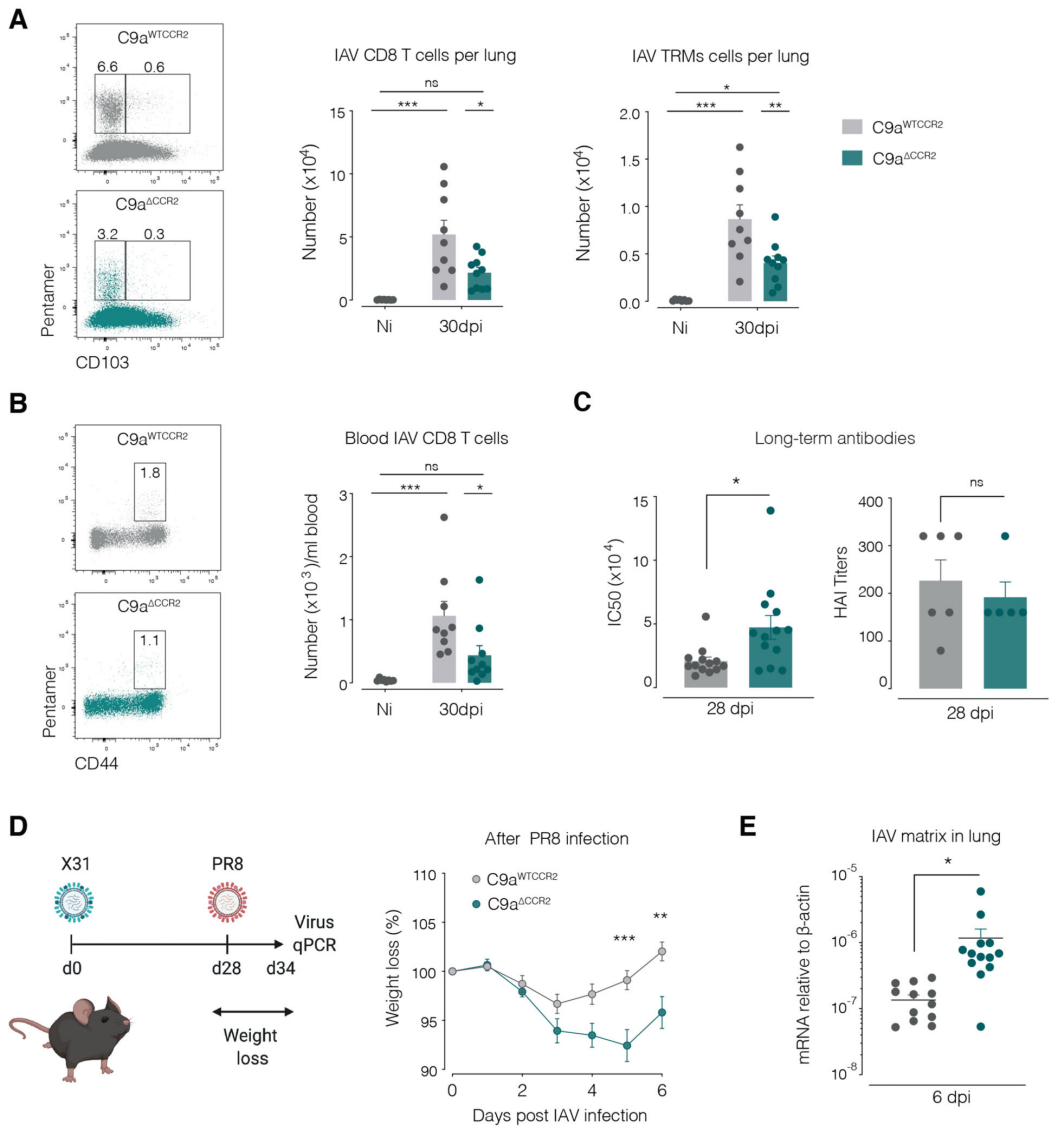
*C9a^ΔCCR2^* mice are susceptible to re-infection with heterologous Influenza. (**A**, **B**) Number of IAV NP-specific pentamer^+^ CD8^+^ T cells in lung (A) or in blood (B) during the memory phase of the response to IAV infection. Lung T cells gated as in Figure 6 were separated into CD103^-^ (left) and CD103^+^ (right) populations. (**C**) IgG against haemagglutinin as measured by ELISA IC50 or HAI titres at 28 days after X31 infection. (**D**) Rechallenge schematic and weight curves for *C9a^WTCCR2^* (grey) and *C9a^ΔCCR2^* (teal) mice during recall IAV challenge using PR8 strain. (**E**) RT-qPCR for mRNA encoding IAV matrix protein in lungs. Data show responses in *C9a^WTCCR2^* (grey) or *C9a^ΔCCR2^* (teal) mice at the specified time points after PR8 infection. Each dot represents one mouse (*n* = 7-10) and data were pooled from 2 experiments. Error bars represent SEM. Statistical test was done using one-way ANOVA test in A, B, E (weight loss) and *t*- test in C, F (IAV matrix). * *p* ≤ 0.05, ** *p* ≤ 0.01, *** *p* ≤ 0.001. Not significant (ns) or when not indicated in E.

cDCs are also key for the induction of long term memory responses to viral infection, including generating tissue resident memory T cells (TRMs) (30). When examining T cell memory formation in *Clec9a^ΔCCR2^* mice, we found that 1 month after IAV infection the number of IAV-specific CD8^+^ T cells in circulation and in the lungs, including canonical CD103^+^ TRMs, was reduced compared to *Clec9a^WTCCR2^* controls (Fig. 7A, B). In contrast, the quantity and neutralising ability of anti-IAV antibodies were either unaffected or, if anything, slightly increased in *Clec9a^ΔCCR2^* mice (Fig. 7C). Importantly, re-infection of mice with a heterologous IAV strain (PR8; H1N1) (Fig. 7D), which is not neutralised by antibodies against X31 (H3N2), induced increased acute weight loss in *Clec9a^ΔCCR2^* mice compared to *Clec9a^WTCCR2^* controls (Fig. 7D), which was accompanied by reduced ability to clear the re-challenge virus (Fig. 7E). Together, these data indicate that expansion of the lung cDC network by pre-cDCs recruited via CCR2 to infection foci is necessary to support the effective generation of effector and memory T cell responses against IAV that protect from re-infection (29).

## Discussion

cDCs are positioned at barrier sites to ensure the early detection of infection. Upon pathogen encounter, they migrate to draining lymph nodes to prime T cells and die 1-3 days thereafter (1, 31). In order to support and diversify T cell priming, the influx of tissue-derived mig-cDCs carrying antigen needs to be sustained (30, 32). However, the basal density of cDCs in tissues is low and it is therefore reasonable to speculate that cDC numbers need to increase at the site of pathogen challenge in order to meet dLN demand for mig-cDCs. During inflammation, monocytes can differentiate into cells that have many cDC features, but it is clear that such monocyte-derived DC-like cells are distinct from those that arise from regular cDCpoiesis and cannot substitute for them, for example in anti-viral or anti-tumour immunity (1). We have previously shown that local proliferation of cDCs and pre-cDCs is decreased during IAV challenge (7) and acute demand for cDCs at sites of infection therefore requires increased recruitment of cDC progenitors. Here, we show that, in the context of IAV infection in mice, this increased local demand is met by recruiting pre-cDCs to lung infection foci in a CCR2-dependent manner. The terminal differentiation of recruited pre-cDCs sustains cDC accumulation next to the antigen source, offsetting cDC emigration to draining lymph nodes and maintaining antigen sampling to induce robust T cell immunity.

CCR2 is generally considered a monocyte marker, although it has been previously reported on some cDC2s, especially inf-cDC2s (5, 23, 33). Ablation of CCR2 in cDCs in *Clec9a^ΔCCR2^* mice, could, in theory, impact cDC migration from lung infection foci to lymph nodes. However, we show that activated lung CCR7^+^ cDCs downregulate CCR2 as they initiate migration to mdLN, presumably to become desensitized to the high levels of CCR2 ligands in lung tissue that would oppose such migration. As for possible CCR2-dependent mobilization of differentiated cDCs from BM to infected lungs, we cannot formally exclude it due to the lack of genetic models to specifically target cDCs without affecting their precursors (or vice versa). However, tissue seeding with cDCs is mediated by migratory precursors (pre-cDCs) rather than by differentiated cells (11). Our data indicate that expression of CCR2 by pre-cDCs does not affect steady-state colonisation of the lungs but is responsible for the increased seeding observed in inflammatory conditions. Consistent with that notion, CCR2 expression is upregulated in BM pre-cDCs during IAV infection, as are CCR2 ligands in the lung, suggesting the existence of an axis where both the receptor and ligand are induced at distant locations to attract target cells to the site of infection. The finding that CCR2 can mediate pre-cDC recruitment suggests caution is warranted when using CCR2-deficient mice or anti-CCR2 antibodies to interrogate the role of monocytes in immunity and inflammation (34, 35).

Interestingly, recent work has revealed a role for CCR2 in increasing numbers of cDCs in lymph nodes draining sites of intramuscular immunisation with AS01-adjuvanted antigens (36). Global loss of CCR2 resulted in decreased CD4^+^ T cell, CD8^+^ T cell and antibody responses to the antigens, suggesting a specific role for inf-cDC2s (36). By restricting receptor loss to the cDC lineage, we find that CCR2 is required for lung increases not only in inf-cDC2s but also in cDC1s and cDC2s in a model of IAV infection and that this is essential for effective T cell-based but not humoral immunity. Which cDC type contributes predominantly to the CCR2-dependence of the T cell response remains unclear although CD8^+^ T cell priming to IAV in mice has been proposed to be particularly dependent on cDC1s (6, 37).

It is likely that not all emergency cDCpoiesis is exclusively dependent on CCR2. Indeed, RNAseq analysis revealed that pre-cDCs in BM also express *Ccr1, Ccr5* and *Cxcr4*, the ligands for which are also increased in mouse lungs during IAV infection. Those receptors could potentially contribute to pre-cDC recruitment to IAV-infected lungs as accumulation of the cells was not fully abrogated in *Clec9a^ΔCCR2^* mice (although this might also reflect incomplete penetrance of the recombination event (15)). The expression of other chemokine receptors could also explain why CCR2-deficiency does not prevent the increase in lung pre-cDCs numbers after *N*. *brasiliensis* infection. Interestingly, the egress of pre-cDCs from BM into blood in IAV-infected mice is not affected by CCR2-deficiency, which is in contrast to monocytes (39). It is therefore likely that BM exit of pre-cDCs and subsequent homing to tissues involves distinct signals and context-specific chemokines, both in steady-state and in emergency cDCpoiesis. The reliance on multiple mechanisms allied to the redundancy of the chemokine system (38) likely ensures robustness and increases resistance to pathogen interference with cDC-driven immunity.

Lung architecture presumably prevents fully-differentiated cDCs scattered around airways and alveoli from migrating through the tissue to coalesce at foci of infection. Therefore, as for other lung resident cells such as macrophages, increasing local cell density requires acute focal recruitment of precursors from the blood (39). Consistent with this hypothesis, we were able to visualise pre-cDCs clustering around IAV infected foci. Our data suggest a model in which challenge of the respiratory tract with IAV rapidly communicates to the BM a need for emergency cDCpoiesis. Pre-cDCs acutely released from BM (7) in a CCR2-independent manner circulate via blood and extravasate from lung capillaries adjacent to foci of infection in response to CCR2 ligands. These include CCL2 produced by CD64^high^ monocytes that were recruited by a prior wave of CCL2 expression by epithelial cells (19). Pre-cDCs then differentiate locally at sites of infection to expand the number of cDCs in close proximity to the antigen source. Newly-generated cDCs expressing CCR2 are retained at foci of infection by CCL2 until they contact virus or virus-infected cells, which act as a source of antigen and activation signals (1). Activation leads to a switch in chemokine receptor expression characterised by upregulation of CCR7 and downregulation of CCR2, allowing the antigen-laden cells to migrate to mdLNs (1, 31). This is dispensable for the antibody response but sustains T cell priming and supports generation of a productive memory response that confers T cell mediated cross-strain protection to heterologous IAV strains. Additional studies will be necessary to establish to what extent pre-cDC back-up is important for immunity to other immune challenges. Such studies could reveal whether emergency cDCpoiesis is an integral part of robust immune responses to infection that could be exploited in vaccine design or, conversely, inhibited in some settings in order to dampen immunopathology caused by T cells.

## Methods

### Study design

The aim of this study was to examine the requirement for cDC expansion to achieve effective adaptive immunity against respiratory viruses. We used IAV as a widely-studied respiratory virus and the mouse as model organism. Mouse experiments were planned in accordance with the principles of the 3Rs (Replacement, Reduction and Refinement) following UK Home Office guidelines. The X31 strain of IAV (H3N2) was used as a mouse-adapted strain for infections and the PR8 strain (H1N1) as a re-infection virus to look at T cell-mediated protection. Different immune cells were examined in lung, blood and bone marrow by flow cytometry and fluorescence confocal microscopy. In addition, chemokines, viral content and antibodies were measured using techniques such ELISA, CBA and qPCR. T cell responses were monitored by ex vivo re-stimulation with defined IAV epitopes. All experiments were performed at least twice. During analysis no individual data points were excluded under any circumstances other than technical failure to process the sample.

### Mice

*Ccr2^fl-eGFP^* (21), *Clec9a^Cre^* (here sometimes abbreviated *C9a^Cre^*) (15), *Flt3l^-/-^* (Taconic Biosciences), *Rosa26^LSL-tdTomato^* (abbreviated *R26^LSL-tdTomato^*; The Jackson Laboratory) and C57BL/6J mice were bred at The Francis Crick Institute in specific pathogen-free conditions. All genetically modified mouse lines were backcrossed to C57BL/6J. Six to twelve-week-old male and female mice were age and sex-matched in all experiments. Sample sizes were determined so as to include the minimum number of mice necessary to achieve statistical robustness when assessing differences in pre-cDC/cDC numbers caused by influenza A virus infection (7). Mice were not randomized in cages, but each cage was randomly assigned to a treatment group. Investigators were not blinded to mouse identity during necropsy and sample analysis. Male and female mice were used to perform the experiments. However, we did not observe differences between sexes. All animal experiments were performed in accordance with national and institutional guidelines for animal care.

### Pre-cDC differentiation assays

Flt3L-driven differentiation of pre-cDCs was carried out by culturing 2 × 10^4^ OP9-DL1 (18) cells into twelve-well plates in RPMI medium supplemented with L-glutamine (Gibco), penicillin-streptomycin (Gibco), non-essential amino acids (Gibco), 10% FCS (Sigma) and β-mercaptoethanol (Gibco). The following day, 1-5 × 10^3^ sorted cells were added to the OP9-DL1 monolayer after removing the medium and replacing it with fresh medium containing 300 ng/ml of mouse Flt3L. Progeny cells were assessed by flow cytometry three days later. cDC differentiation was assessed by MHC-II upregulation, whereas PC differentiation was quantified by the expression of B220 and SiglecH. cDC1s were defined as XCR1^+^ and cDC2s as SIRPα^+^.

### Infection

Mice were anesthetised by isoflurane inhalation and were infected intranasally with 35,000 tissue culture infectious doses 50 (TCID50) of influenza A X31 (H3N2) in 30μl PBS. For heterosubtypic challenge, mice were infected with X31 influenza at the same dose followed by i.n. administration of 10,000 TCID50 of H1N1 PR8 IAV (in 30μl PBS), 28 days after primary infection. Mice were monitored daily for weight loss and signs of infection. *N*. *brasiliensis* was maintained by serial passage through rats, as described previously (24). Mice were infected subcutaneously with 250 *N*. *brasiliensis* L3 larvae.

### Adoptive transfers

Bone marrow was isolated from the legs, arms, hipbone, sternum and spinal cord of CD45.1 C57BL/6J mice at 1 day post infection with IAV. CD11b^hi^, CD11b^lo^ and CD11b^-^ cells and monocytes were sorted as indicated in Fig. 1A (gates 4) and Fig.S2A. 70,000-150,000 cells were injected intravenously into CD45.2 C57BL/6J mice 1dpi with IAV. Lung and mdLN cells were analysed 4 days later (5dpi).

### In vitro re-stimulation

For IAV-specific T cells, 5 x10^5^ freshly-isolated mdLN or lung cells were plated and re-stimulated with 1μM, 1nM or 1pM of IAV PA224-233, NP366-373 or NP276-290 peptides (all from Crick Peptide Synthesis facility, Table S4) at 37°C and 5% CO_2_.in RPMI with 10% FCS, 1uM β-mercaptoethanol and 1% PenStrep Glutamine. For *N*. *brasiliensis-specific* T cells, mdLN cells were re-stimulated with parasite extract (1 μg/ml) or Dynabeads™ Mouse T-Activator CD3/CD28 (8 million per well, Gibco). After 72h, cells were lysed by freezing the plate at -80°C. Supernatants were analysed for IFN-γ, IL-4, IL-5 or IL-13 content by CBA (as for CCL2 quantification, see below; BD Biosciences)

### Preparation of single cell suspensions

Spleens, mdLNs, iLNs and lungs were cut into small pieces and digested with Collagenase VIII (1mg/ml, Sigma) or VI (400U/ml, Worthington) and DNase I (0.4mg/ml, Roche) in RPMI for 15-30 min (spleen and LNs) or 20-60 min (lung) at 37°C. Digested tissues were strained through a 70μm cell strainer (BD Bioscience) and washed with FACS buffer (3% foetal calf serum, 5mM EDTA in PBS). For lung, leukocytes were enriched by Percoll gradient centrifugation (GE Healthcare) as previously described (7). For BM, femur and tibia extremities were cut and spun for 30s at 10,000 rpm. Cells were resuspended in final volume of 500μl.

### Flow Cytometry analysis

Cells were preincubated with blocking anti-CD16/32 in PBS for 10 min at 4°C and then stained for 20 min at 4°C with antibody cocktail and LIVE/DEAD Fixable Blue Dead Cell Stain Kit (ThermoFisher) in PBS. Lineage (Lin) markers included CD3, Ly6G, SiglecF, B220, CD19, NK1.1, Ly6D and Ter119, unless otherwise specified. Antibodies (Abs) used for flow cytometry are listed in Table S2. PE conjugated pentamer comprising H2-D^b^-ASNENMETM (ProImmune) was used to detect IAV NP-specific CD8^+^ T cells. Data was analysed using FlowJo. Uniform manifold approximation and projection (UMAP) analysis (40) of flow cytometry data were generated on the basis of CD11b, CD11c, CD16/32, CD26, CD43, CD64, CD88, CD135, Sirpα, MHCII, Ly6C, SiglecH and XCR1 expression. Annotation of clusters on the UMAP plots was done by using defining markers for each immune population. The accuracy of our manual gating was confirmed on the UMAPs by overlaying different immune populations identified as shown in Figure 1E and S2A.

### RNA extraction, cDNA synthesis and RT-qPCR

mdLNs and lungs were collected in Trizol and subsequently homogenised in a TissueLyser LT (Qiagen). RNA was isolated using chloroform and precipitated with isopropanol. After washing with ethanol, RNA was resuspended and used to synthesise cDNA using the Superscript II reverse transcriptase (Invitrogen). Quantitative real-time PCR (RT-qPCR) was performed using the TaqMan Universal PCR Master Mix (ThermoFisher) and primers (listed in Table S5). Analysis was performed on a QuantStudio (Thermo Fisher Scientific) using ΔCt quantification. RT-qPCR conditions were as per manufacturer’s instructions.

### RNAseq

For Figure 2E, pre-cDCs were isolated from BM using a BD FACS Aria Fusion sorter. 1-6 × 10^4^ cells were sorted directly into lysis buffer to avoid loss of material. RNA was extracted using the RNeasy Mini Kit (Qiagen). NuGEN Ovation RNA-Seq System (v2) was used for cDNA synthesis followed by NuGEN UltraLow Library System (v2) for library preparation. Samples were normalised to 1 ng RNA for input and the preparation was performed according to the manufacturer’s guidelines. Sequencing was performed on the Illumina HiSeq 4000, with 100 base pair single end reads. After sequencing, samples were normalised and analysed.

### Microscopy

Lungs were removed and the left lobe was fixed in 4% paraformaldehyde (Electron Microscopy Sciences) in PBS overnight at 4°C. Fixed lungs were incubated in PBS with 30% sucrose at 4°C overnight and embedded in Tissue-Tek OCT compound (Sakura) at -80°C. The tissue was cut in a Leica 3050 cryostat to generate 60μm frozen sections. For antibody staining, sections were hydrated in 0.1M TRIS pH 7.4 and blocked for 1h at 25°C in 1% bovine serum albumin (Sigma), 1% normal mouse serum (Invitrogen) and 0.25% Triton TX-100 (Sigma). Sections were first stained overnight at 4°C with rabbit anti-CD64 (clone 027, Sino, 1:1000), rat anti-CD45R/B220 biotin (RA3-6B2, BD Pharmingen, 1:100), rat anti-I-A^b^ biotin (M5-114.14.2, BD Pharmingen, 1:100) and anti-IAV NP+M FITC (Oxoid, 1:100) diluted in blocking buffer. Sections were subsequently stained with anti-FITC AF647 (1F8-1E4, Jackson ImmunoResearch, 1:400) and anti-rat IgG AF594 (Invitrogen, 1:400) or anti-rabbit IgG AF594 (Invitrogen, 1:400). Sections were counterstained in Hoechst 33342 solution (ThermoFisher, 1:500) and mounted in RapiClear 1.47 (Sunjin Lab). Antibodies used for confocal microscopy are listed in Table S4. Imaging was performed in a Zeiss LSM 880 inverted confocal microscope with a 25x oil immersion objectives. Sequential excitation of fluorophores at 405nm, 488nm, 561nm and 633nm was provided by a combination of argon and helium lasers. Tile scans were acquired covering the entire surface area of the section at a step size of 3μm and a pinhole of 1 Airy unit. Images were acquired with 512x512 pixel resolution with a line averaging of 4. Tile stitching was performed using Zen software (Zeiss).

### Pre-cDC localisation analysis

Three dimensional volumetric surfaces corresponding to lung pre-cDCs, IAV^+^ and lineage^+^ cells (lin = CD64, B220, I-A^b^) were generated in Imaris 9.2 (BitPlane). Thresholding for pre-cDC surface generation was based on eGFP intensity and then an additional filter was added to exclude lin^+^ cells. Statistics were exported and analysed using GraphPad Prism. Surface coordinates were exported and plotted in two dimensions (X and Y). Non-parametric probability density functions (PDFs) for pre-cDCs and IAV^+^ cells in a given lung section were generated using the ksdensity function from the Statistics and Machine Learning toolbox in MATLAB (MathWorks). Boundary correction of reflection and a bandwidth of 100 were selected; additional bandwidths were tested for robustness. Statistical distance metrics (Jensen Shannon distance and Hellinger distance) for continuous distributions were used to compare the similarity between pre-cDC and IAV PDFs and determine pre-cDC/IAV co-localization (42). All measures are bounded between zero and one. To assess the randomness of the distribution of pre-cDCs and IAV^+^ cells, these distance metrics were used to compare pre-cDC and IAV PDFs to tissue specific uniform distributions generated for each lung section based on saturated Hoechst staining. Each particular point within the stained tissue has an equal (uniform) probability of a pre-cDC or IAV^+^ cell being located there.

### Haemagglutination inhibition assay

Haemagglutination inhibition (HAI) activity was evaluated using previously described protocols (41). IAV (X31 strain) diluted in PBS to give 8 haemagglutination activity units and serum samples (diluted 1:5 in Seiken Receptor Destroying Enzyme (Cosmos Biomedical Ltd.) followed by 1:2 serial dilutions in PBS) were incubated in V-bottom 96 microtitre plates for 30 min at room temperature. Turkey red blood cells (0.75% (v/v); supplied by PHE, Colindale) were added to the antibody–virus mixture, mixed gently, and incubated for 30 min at room temperature. Wells were scored visually for haemagglutination. HAI titres were recorded as the reciprocal of the highest serum dilution that prevented haemagglutination.

### Anti-haemagglutinin ELISA

Purified haemagglutinin (H3N2, strain X31, a gift from J. Skehel) was coated in high-binding 96-well microtitre plates (Nunc) and after overnight incubation at 4 °C, plates were blocked with PBS plus 3% (v/v) FSC for 2 h. After blocking, plates were incubated for 2 h with serially diluted serum samples. Initial dilution of sera was 1:50 followed by 1:2 serial dilutions when samples were collected 0-5 dpi with IAV and 1:100 followed by 1:3 serial dilutions when samples were collected 28 dpi with IAV. Serum samples were washed, followed by HRP-conjugated goat anti-mouse IgG (Biorad, 170-6515, 1h; 1:3000) or anti-mouse IgM (Biorad, STAR86P, 1h; 1:1000). Plates were developed using TMB (3,3’,5,5’-Tetramethylbenzidine, Invitrogen) and reactions were stopped with the addition 0.18M sulphuric acid. Absorbance at 450 nm was immediately recorded and background absorbance from negative control samples was subtracted. Titre (IC50) was calculated as the reciprocal of the dilution that gave the half-maximal absorbance value. Alternatively, area under curve (AUC) was calculated using GraphPad Prism.

### Quantification of chemokines in lung homogenates and BALF

Lung homogenates were generated by mechanical tissue lysis in PBS with Halt protease inhibitor cocktail (ThermoFisher) and supernatants were stored at −80°C. For isolation of fluid from the bronchoalveolar space, mice were sacrificed and bronchoalveolar lavage was carried out by flushing with 2 × 0.6ml cold PBS using a needle inserted into the trachea. Cells were removed by centrifugation at 800g for 8 min at 4°C and supernatants were used for analysis. Chemokines and cytokines were analysed using the mouse chemokine array ARY020 kit (R&D). Altenatively, mouse CCL2 Flex Set (BD Biosciences) was used for CCL2 quantification by cytometric bead array (CBA, BD Biosciences) in lung homogenates and BALF. Events were acquired on an LSRFortessa or FACSymphony flow cytometer (BD Biosciences). Analysis was carried out using FlowJo 10 software (TreeStar Inc). Flow Cytometry Standard (FCS) files were exported from FlowJo for analysis by Uniform Manifold Approximation and Projection (UMAP) performed in R (40).

### Statistical Analysis

Statistical analyses were performed using GraphPad Prism software (GraphPad) or MATLAB (Mathworks). Results are depicted as means +/- SEM. The statistical test used is specified in each figure legend. When Student’s *t* test was perfomed, a two-side level was used and all data were checked for normality. When ANOVA was used, Tukey correction was performed.

## Supplementary Material

Data file S1

Fig. S1

## Data Availability

RNA sequencing data has been deposited in GEO under accession number XX. All other data needed to evaluate the conclusions in the paper are present in the paper or the Supplementary Materials.

## One-sentence summary

Effective T cell-dependent immunity to influenza A virus requires recruitment of cDC progenitors to foci of infection via CCR2
