# Supplementary material for: Recruitment of dendritic cell progenitors to foci of influenza A virus infection sustains immunity
Source: Sci Immunol. Author manuscript; Available in PMC 2021 Nov 22. (PMC7612017; doi:10.1126/sciimmunol.abi9331)
Supplement: Fig. S1 [file EMS138719-supplement-Fig__S1.pdf]

List of supplementary materials

Figure S1. Cell fate of different myeloid populations after transfer into IAV-infected hosts.

Figure S2. cDC population dynamics during IAV infection.

Figure S3. IAV infection in *C9a*$^{\Delta CCR2}$ mice.

Figure S4. *N. brasiliensis* infection in *C9a*$^{\Delta CCR2}$ mice.

Figure S5. Validation of microscopy approach to imaging lungs from *C9a*^{cre} *Ccr2*$^{fl\text{-}eGFP}$ mice.

Figure S6. Normal immune cell composition in lymphoid organs from *C9a*$^{\Delta CCR2}$ mice.

Figure S7. T cell response kinetics during type 1 vs. type 2 infection.

Table S1. Cell recovery and subset differentiation of Flt3L cultures

Table S2. Antibodies used in flow cytometry

Table S3. Antibodies used in microscopy

Table S4. IAV peptides for *in vitro* re-stimulation

Table S5. Oligonucleotides for qPCR

Data file S1. Raw data file (Excel spreadsheet)

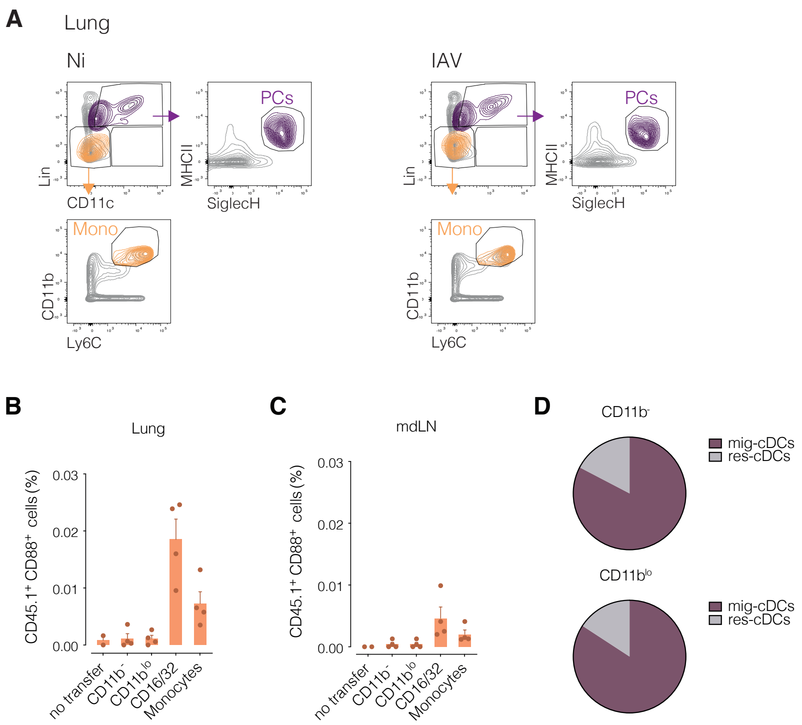

Figure S1. Cell fate of different myeloid populations after transfer into IAV-infected hosts.

(**A**) Gating strategy for identifying classical Ly6C$^+$ monocytes and PCs in lungs before and after (5dpi) IAV infection. (**B, C**) Percentage of CD45.1$^+$ CD88$^+$ cells recovered from lungs **(B)** or mdLNs **(C)** of CD45.2 recipient mice after transferring the CD45.1 cells listed on the x-axis. From experiment depicted in Figure 1J, K. (**D**) Mig-cDCs and res-cDCs from experiment depicted in Figure 1K.

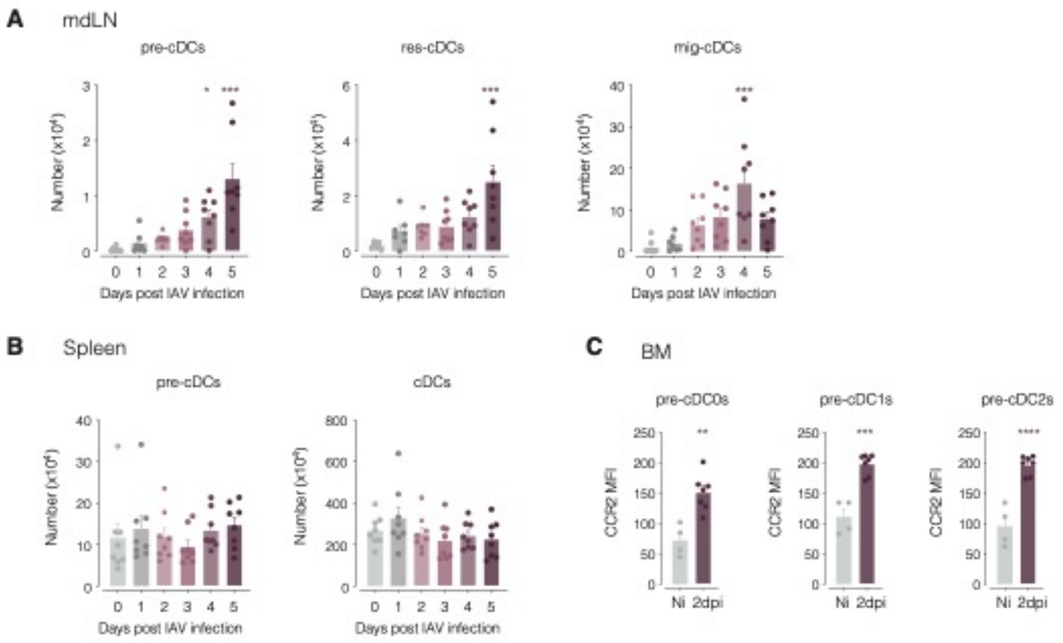

Figure S2. cDC population dynamics during IAV infection.

(A, B) Numbers of pre-cDCs and cDCs (resident cDCs ($MHCII^{lo}$, $CD11c^{hi}$) and migratory cDCs ($MHCII^{hi}$, $CD11c^{lo}$) in **(A)** mdLNs and of pre-cDCs and cDCs in **(B)** spleens from mice infected (purple) or not (Ni, grey) with IAV. **(C)** CCR2 mean fluorescence intensity in BM pre-cDC subsets from Ni or mice infected with IAV and analysed 2dpi. Each dot represents one mouse (*n* = 8) and data are pooled from two independent experiments. One-way ANOVA test was used in B, C and unpaired t-test in D. * $p \leq 0.05$, ** $p \leq 0.01$, *** $p \leq 0.001$. Not significant when not indicated.

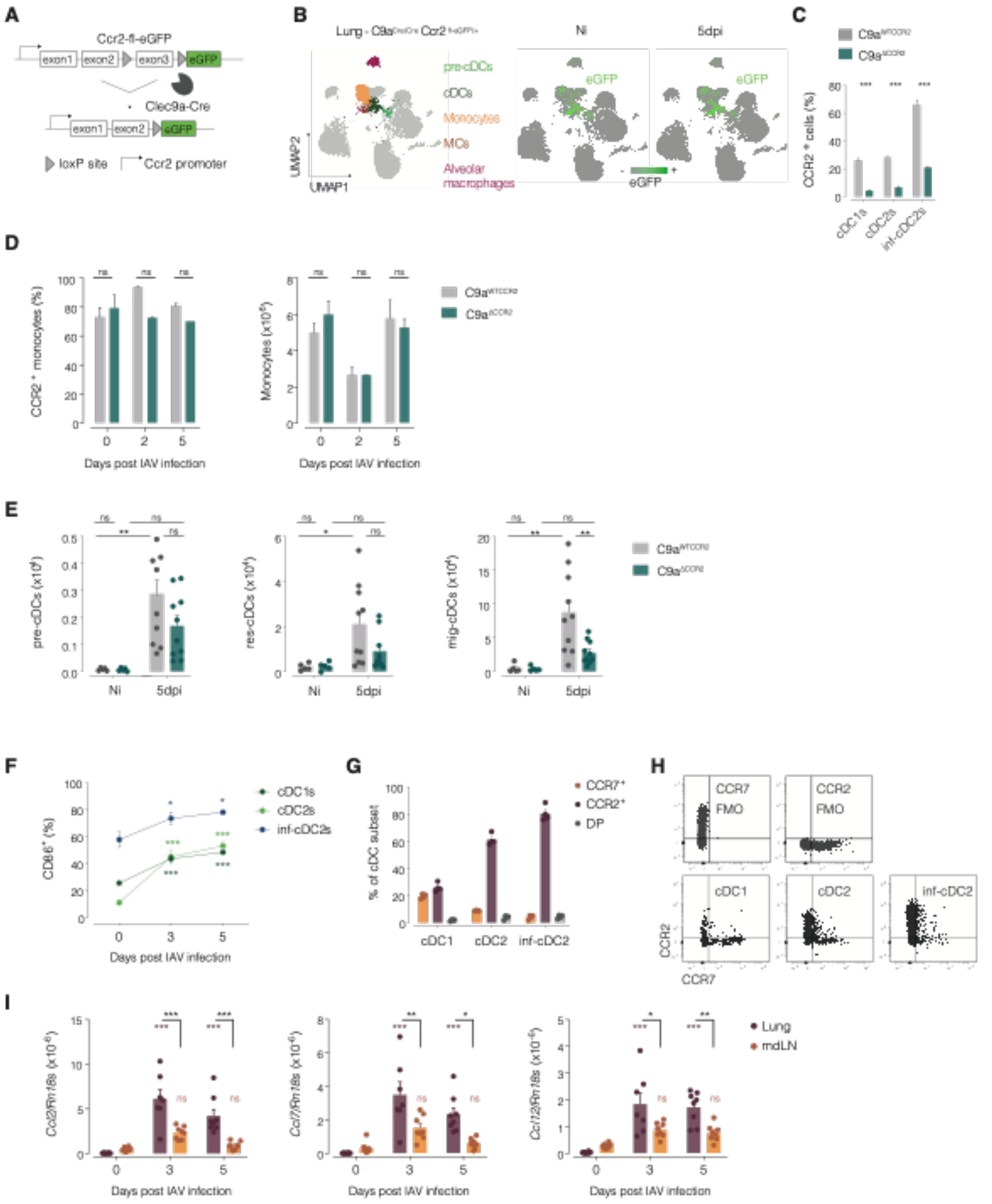

Figure S3. IAV infection in *C9a*$^{\Delta CCR2}$ mice.

(A) Schematic depiction of the genetic recombination event in *Clec9a*$^{Cre/Cre}$ *CCR2*$^{fl\text{-}eGFP/fl\text{-}eGFP}$ mice. **(B)** UMAP analysis of flow cytometry data showing CD45^{+} cells overlaid with different immune populations as identified with manual gating (left panel) and eGFP intensity in samples (right and center panels) from lung of *C9a*$^{Cre/Cre}$ *Ccr2*$^{fl\text{-}eGFP/+}$ mice at steady state and 5dpi with IAV. **(C)** CCR2^{+} cells

in lung cDC subsets from *C9a*^{WTCCR2} (grey) and *C9a*$^{\Delta CCR2}$ (teal) mice 5dpi with IAV. **(D)** CCR2 expression (left panel) and total number (right panel) of monocytes in BM from *C9a*^{WTCCR2} (grey) and *C9a*$^{\Delta CCR2}$ (teal) mice at indicated days after IAV infection. **(E)** Number of mdLN pre-cDCs, resident cDCs (MHCII^{lo}, CD11c^{hi}) and migratory cDCs (MHCII^{hi}, CD11c^{lo}) from naïve and IAV-infected *C9a*^{WTCCR2} (grey) or *C9a*$^{\Delta CCR2}$ (teal). **(F)** Percentage of CD86^{+} cells in each cDC subset at specified days post IAV infection. **(G)** Percentage of the indicated cDC subsets that is positive for CCR2, CCR7 or both (double positive, DP) during IAV infection. **(H)** Representative plots from indicated cDC subsets in E, including fluorescence minus one (FMOs) controls. **(I)** *Ccl2*, *Ccl7* and *Ccl12* transcripts in whole lung or mdLN homogenates at different times post infection with IAV. Each dot represents one mouse (*n* = 5-10) and data are from 2 pooled experiments. Statistical analysis was done using a two-way ANOVA test. *p* values are indicated. * $p \leq 0.05$, ** $p \leq 0.01$, *** $p \leq 0.001$. Not significant (ns).

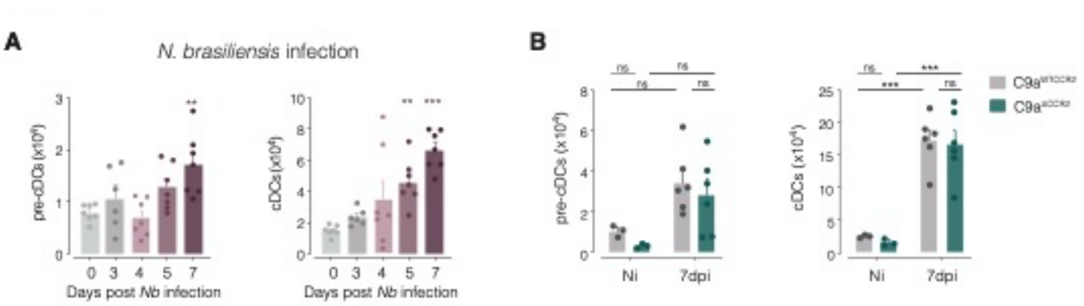

Figure S4. *N. brasiliensis* infection in *C9a*$^{\Delta CCR2}$ mice.

(**A**) Number of pre-cDCs (left) or cDCs (right) in lungs from C57BL/6J mice at different times post-infection with *N. brasiliensis*. (**B**) Number of pre-cDCs (left) and cDCs (right) in lungs of *C9a*^{WTCCR2} (grey) and *C9a*$^{\Delta CCR2}$ (teal) mice 7dpi with *N. brasiliensis.* Each dot represents one mouse (n = 3-7) and data are from 2 pooled experiments. Statistical analysis was done using a one-way (A) or two-way (B) ANOVA test. p values are indicated. * $p \leq 0.05$, ** $p \leq 0.01$, *** $p \leq 0.001$. Not significant (ns).

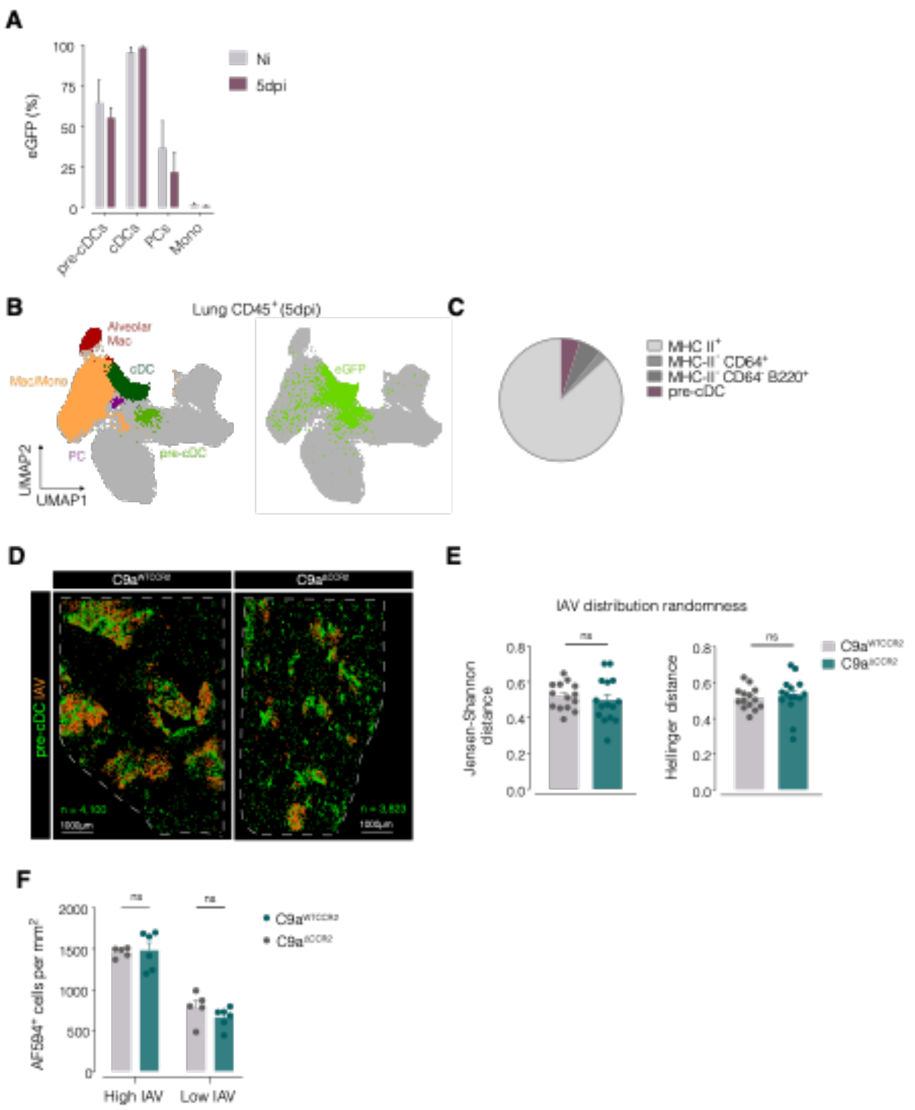

Figure S5. Validation of microscopy approach to imaging lungs from *C9a*^{Cre} *Ccr2*$^{fl\text{-}eGFP}$ mice.

(A) Percentage of eGFP^{+} cells in the selected populations from *Clec9a*$^{Cre/Cre}$ and *Ccr2*$^{fl\text{-}eGFP}$ (heterozygous or homozygous) lungs from Ni mice or 5dpi with IAV determined from flow cytometry data. **(B)** UMAP analysis of flow cytometry data depicting lung immune cell populations (left) and eGFP^{+} cells (right) in a *C9a*^{WTCCR2} mouse 5 days post IAV infection. **(C)** Frequency of lung eGFP^{+} pre-cDCs and eGFP^{+} cells expressing MHC class II, CD64 or B220 in *C9a*^{WTCCR2} mice 5 days post IAV infection (n = 3) analysed by flow cytometry. **(D)** 3D projection from representative lung sections from *C9a*^{WTCCR2} or *C9a*$^{\Delta CCR2}$ mice 5 days post infection with IAV, stained anti-IAV M+NP and for lineage markers (anti-MHC class II, anti-CD64, anti-B220) (left). Images shows the localization of pre-cDCs (eGFP^{+} AF594^{-}) and IAV^{+} cells (M+NP staining) based on the coordinates of volumetric surfaces generated using Imaris software. Dashed lines delineate the outline of imaged lung tissue based on Hoechst staining. n indicates the number of

pre-cDC surfaces in each section. **(E)** IAV M+NP distribution randomness scores in lung tissue sections from $C9a^{WTCCR2}$ and $C9a^{\Delta CCR2}$ mice calculated by Jensen-Shannon and Hellinger distance metrics. **(F)** Microscopic quantification of $AF594^{+}$ cells in areas with high or low IAV abundance measured by staining with M+NP from $C9a^{WTCCR2}$ or $C9a^{\Delta CCR2}$ mice 5 days post infection. Each dot corresponds to the analysis of one image from a lung section (*n* = 29 images from 10 mice). Statistical analyses were performed using an unpaired t-test. * $p \leq 0.05$, ** $p \leq 0.01$, *** $p \leq 0.001$. Not significant (ns).

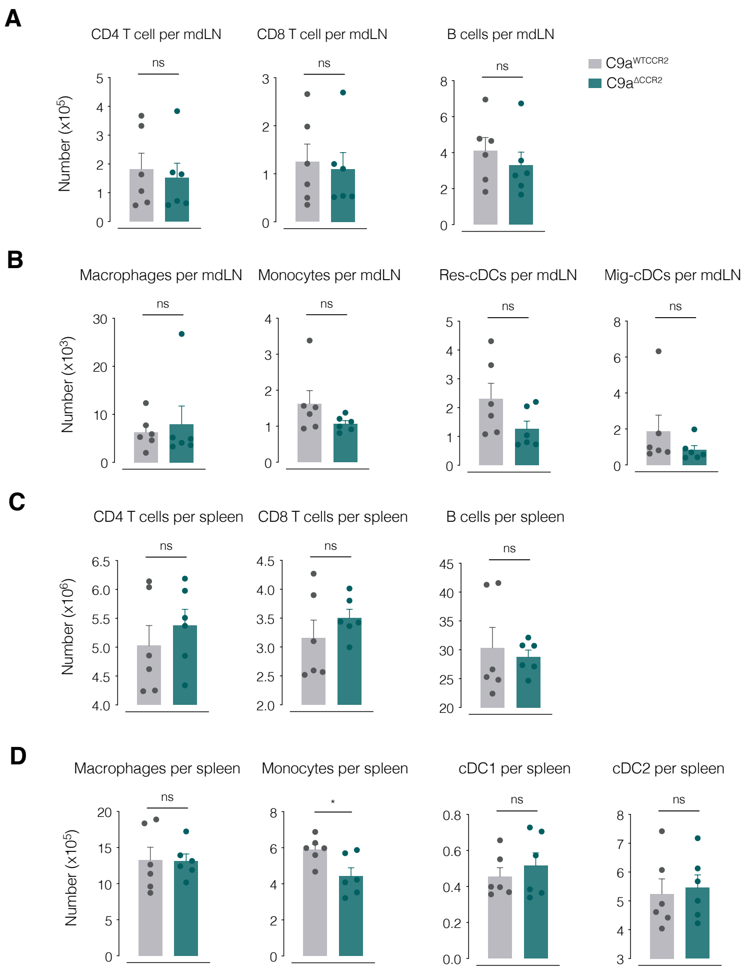

Figure S6. Normal immune cell composition in lymphoid organs from *C9a*$^{\Delta CCR2}$ mice.

(**A**-**D**) Flow cytometry analysis of the number of cells of T and B cells **(A, C)** or monocytes, macrophages and cDCs **(B, D)** in mdLNs **(A, B)** and spleen **(C, D)** of *C9a*^{WTCCR2} (grey) and *C9a*$^{\Delta CCR2}$ (teal) mice. Each dot represents one mouse (*n* = 6) and data were pooled from 2 experiments. T cells were gated as live, CD45^{+} cells, B220^{-}, TCRβ^{+} and CD8^{+} or CD4^{+} cells. B cells were gated as live, CD45^{+} cells, B220^{+}, MHC class II^{+} cells. Macrophages were gated as live, CD45^{+} cells, CD64^{high}, Ly6C^{-} cells and monocytes as CD45^{+} cells, CD64^{high}, Ly6C^{+} cells. cDCs were gated as in Fig. S1B. Levels of MHC class II and CD11c were used to define resident (res-cDCs, MHCII^{low} CD11c^{high}) and migratory (mig-cDCs, MHCII^{high} CD11c^{low}) cDCs. Unpaired *t*-test was used to compare the two groups. * $p \leq 0.05$. Not significant (ns).

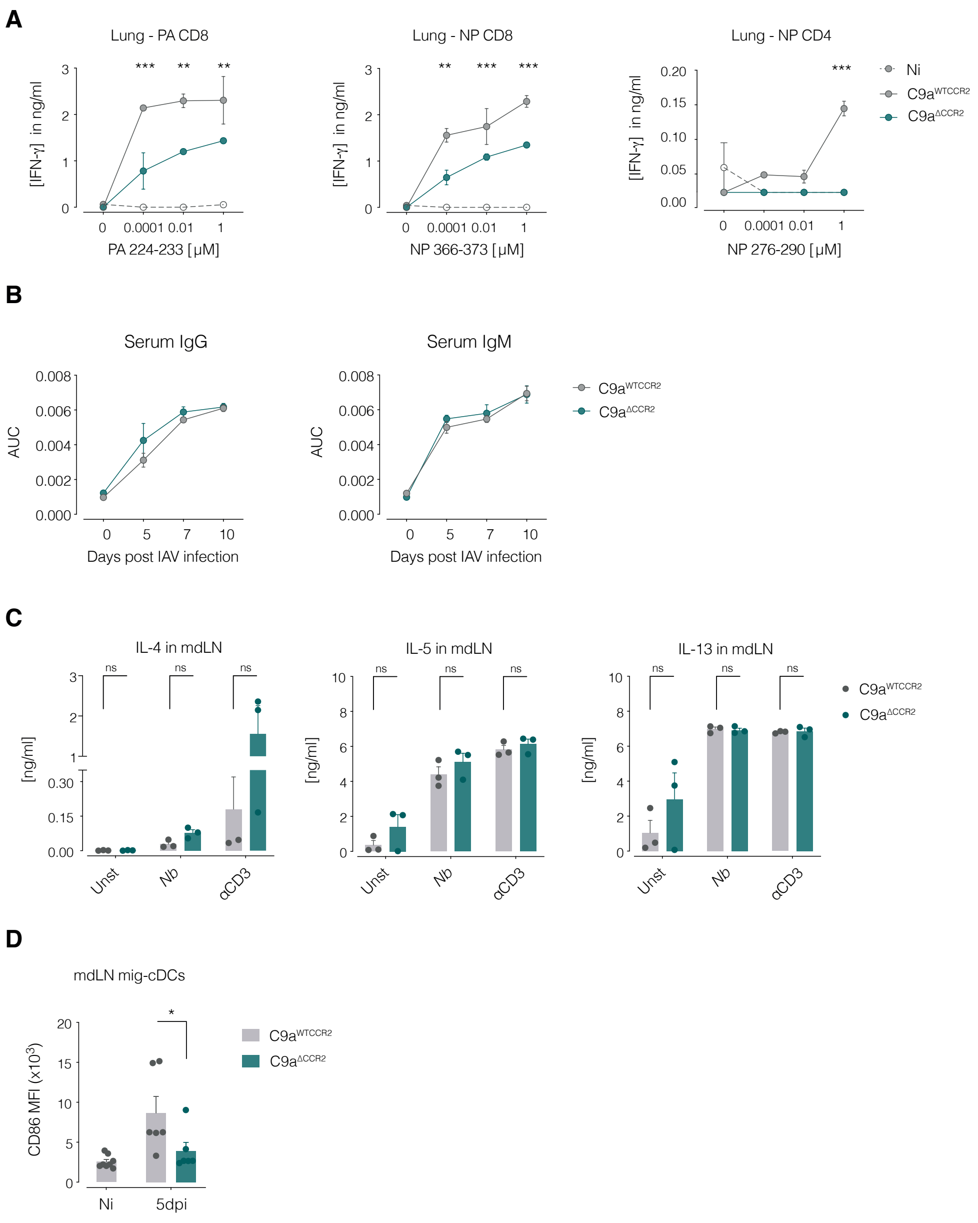

Figure S7. T cell response kinetics during type 1 vs. type 2 infection.

(A) Quantification of IFN-γ in supernatants following 72h of *ex vivo* re-stimulation of lung cells from IAV-infected *C9a*^{WTCCR2} (grey) or *C9a*$^{\Delta CCR2}$ (teal) mice with the indicated doses of IAV PA or NP peptides that can be recognized by IAV-specific H-2^{b}-restricted CD8^{+} or CD4^{+} T cells. Cells from uninfected mice (Ni) were used as a specificity control (open circles). Data are mean from 3 mice from 1 experiment. **(B)** IgG and IgM responses against haemagglutinin as measured by ELISA at indicated time points after X31 infection. AUC, area under curve. **(C)** Quantification of IL-4, IL-5 and IL-13 in supernatants following 72h of *ex vivo* re-stimulation of mediastinal lymph node cells harvested from *C9a*^{WTCCR2} (grey)

or *C9a*$^{\Delta CCR2}$ (teal) mice at 7dpi with *N. brasiliensis* (*N.b.*). Cells were re-stimulated with *N.b.* protein extract or αCD3 microbeads. Each dot represents one mouse (*n* = 3). (**D**) Surface CD86 expression as quantitated by MFI on mig-cDCs in mdLNs of C9a$^{WT\mathit{CCR2}}$ (grey) or C9a$^{\Delta CCR2}$ (teal) mice. Each dot represents one mouse (*n* = 7-9). Data in (A) and (C) are from one experiment, in (B) and (D) are a pool of two experiments. One-way ANOVA statistical test was used to compare groups. * $p \leq 0.05$, ** $p \leq 0.01$, *** $p \leq 0.001$. Not significant (ns).

Table S1. Cell recovery and subset differentiation of Flt3L cultures

	**Sorted population (Fig. S1A)**			
	**CD11b$^-$**	**CD11b^{lo}**	**CD11b^{hi} CD16/32$^+$**	**Ly6D$^+$**
Cell recovery (%)	17.4 ± 7.8	19.5 ± 5.8	8.4 ± 5.1	6.4 ± 2.1
% of cells MHC-II$^+$	91.6 ± 7.4	83.6 ± 12.9	41.2 ± 15.8	36.1 ± 16.5
% of cells B220$^+$ SiglecH$^+$	7.6 ± 5.1	0.8 ± 0.7	1.2 ± 1.7	79.5 ± 15.1

Table S2. Antibodies used in flow cytometry

**Target**	**Fluorophore**	**Clone**	**Company**	**Concentration**
CCR2	PE	475301	R&D	5µg/ml
CCR7	PE-Dazzle	4B12	Biolegend	2µg/ml
CD8-alpha	BV605	53-6.7	Biolegend	10µg/ml
CD11b	APC eFluor780	M1/70	eBioscience	1µg/ml
CD11b	BUV395	M1/70	BD Biosciencee	1µg/ml
CD11b	BV605	M1/70	Biolegend	1µg/ml
CD11c	PerCP Cy5.5	N418	Biolegend	1µg/ml
CD11c	BV650	N418	Biolegend	1µg/ml
CD11c	AF594	N418	Biolegend	1µg/ml
CD11c	BUV805	N418	BD bioscience	2µg/ml
CD16/32	Unconjugated	2.4G2	BD Bioscience	1µg/ml
CD16/32	APC Cy7	93	Biolegend	2µg/ml
CD19	V450	1D3	BD Bioscience	0.5µg/ml
CD25	FITC	7D4	BD Pharmigen	5µg/ml
CD26	BUV737	H194-112	BD Bioscience	2µg/ml
CD43	PeCy7	S11	Biolegend	1µg/ml
CD43	Biotin	1B11	Biolegend	1µg/ml
CD45.2	APC	104	Biolegend	1µg/ml
CD45.2	BV605	104	Biolegend	2µg/ml
CD45.2	BV711	104	Biolegend	2µg/ml
CD45R (B220)	eFluor450	RA3-6B2	eBioscience	2µg/ml
CD45R (B220)	PE	RA3-6B2	BD Bioscience	0.5µg/ml
CD45R (B220)	BUV661	RA3-6B2	BD Bioscience	1µg/ml
CD45R (B220)	BV650	RA3-6B2	Biolegend	1µg/ml
CD62L	APC eF780	MEL14	BD Bioscience	5µg/ml
CD64	PeCy7	X5-4/7.1	Biolegend	2µg/ml
CD64	PE	X5-4/7.1	Biolegend	1µg/ml
CD88	APC	20/70	Biolegend	1µg/ml
CD86	BV711	GL1	BD Bioscience	1µg/ml
CD103	PE	2E7	Biolegend	4µg/ml
CD135	PerCP eF710	A2F10	eBioscience	2µg/ml
CD172-alpha	BV510	P84	Biolegend	1µg/ml
CD172-alpha	AF647	P84	BD Bioscience	1µg/ml
F4/80	BUV563	T45-2342	BD Bioscience	2µg/ml
FceR1a	BV711	MAR-1	BD Bioscience	1µg/ml
I-A/I-E (MHCII)	AF700	M5/114.15.2	eBioscience	1µg/ml

Ly6G	BUV805	1A8	BD Bioscience	1µg/ml
Ly6G	BV421	1A8	Biolegend	1µg/ml
Ly6C	BV785	HK1.4	Biolegend	0.2µg/ml
Ly6D	eF450	49-H4	eBioscience	0.5µg/ml
NK1.1	Pacific Blue	PK136	Biolegend	1 µg/ml
Siglec H	BUV615	440c	BD bioscience	1µg/ml
Siglec F	BV421	E50-2440	BD bioscience	1µg/ml
Ter-119	Pacific Blue	TER-119	Biolegend	2µg/ml
TCR β	APC Cy7	H57-597	Biolegend	1µg/ml
Pentamer NP	PE	ASNENMETM	Proimmune	2.5µg/ml
XCR1	BV650	ZET	Biolegend	1µg/ml

Table S3. Antibodies used in microscopy

**Target**	**Conjugate**	**Origin**	**Clone**	**Company**	**Concentration**
CD45R/B220	Biotin	Rat	RA3-6B2	BD Pharmingen	5µg/ml
CD64	Purified	Rabbit	027	Sino Biological	0.75µg/ml
MHC-II (I-A/I-E)	Biotin	Rat	M5-114.15.2	BD Pharmingen	5µg/ml
IAV NP and M protein	FITC	Unknown	Unknown	Oxoid	1:100
Rat IgG (H+L)	AF594	Goat	Polyclonal	Invitrogen	5 µg/ml
Rabbit IgG (H+L)	AF594	Goat	Polyclonal	Invitrogen	5 µg/ml
FITC	AF647	Mouse	1F8-1E4	Jackson ImmunoResearch	4 µg/ml

Table S4. IAV peptides for *in vitro* re-stimulation

**Peptide**	**Sequence**
PA 224-233	SSLENFRAYV
NP 366-373	ASNENMET
NP 276-290	LPACVYGPAVASGYD

Table S5. Oligonucleotides for qPCR

**Target**	**Primer**	**Sequence**
X31 matrix protein	Fwd	AAGACCAATCCTGTCACCTCTGA
X31 matrix protein	Rev	CAAAGCGTCTACGCTGCAGTCC
X31 matrix protein	Taqman probe	TTTGTGTTCACGCTCACCGT
Beta actin	Taqman probe	Mm00607939_s1 (ThermoFisher)
Ccl2	Taqman probe	Mm00441242_m1 (ThermoFisher)
Ccl7	Taqman probe	Mm00443113_m1 (ThermoFisher)
Ccl12	Taqman probe	Mm01617100_m1 (ThermoFisher)
Rn18	Taqman probe	Mm03928990_g1 (ThermoFisher)
